# The presence of highly disruptive 16S rRNA mutations in clinical samples indicates a wider role for mutations of the mitochondrial ribosome in human disease

**DOI:** 10.1016/j.mito.2015.08.004

**Published:** 2015-11

**Authors:** Joanna L. Elson, Paul M. Smith, Laura C. Greaves, Robert N. Lightowlers, Zofia M.A. Chrzanowska-Lightowlers, Robert W. Taylor, Antón Vila-Sanjurjo

**Affiliations:** a Institute of Genetic Medicine, Newcastle University, Newcastle upon Tyne NE1 3BZ, United Kingdom; b Centre for Human Metabonomics, North-West University, Potchefstroom, South Africa; c Institute of Medical Sciences, Ninewells Hospital and Medical School, Dundee University, Dundee DD1 9SY, Scotland, UK; d Wellcome Trust Centre for Mitochondrial Research, Institute of Neuroscience, Newcastle University, The Medical School, Newcastle upon Tyne NE2 4HH, United Kingdom; e Newcastle University Institute for Cell and Molecular Biosciences, Newcastle University, The Medical School, Newcastle upon Tyne NE2 4HH, United Kingdom; f Grupo GIBE, Bioloxía Celular e Molecular, Facultade de Ciencias, Universidade da Coruña (UDC), Campus Zapateira s/n, 15071 A Coruña, Spain

**Keywords:** MtDNA, Mitochondrial rRNA, rRNA mutation, Mitoribosome, Mitochondrial disease

## Abstract

Mitochondrial DNA mutations are well recognized as an important cause of disease, with over two hundred variants in the protein encoding and mt-tRNA genes associated with human disorders. In contrast, the two genes encoding the mitochondrial rRNAs (mt-rRNAs) have been studied in far less detail. This is because establishing the pathogenicity of mt-rRNA mutations is a major diagnostic challenge. Only two disease causing mutations have been identified at these loci, both mapping to the *s*mall *su*bunit (SSU). On the *l*arge *su*bunit (LSU), however, the evidence for the presence of pathogenic LSU mt-rRNA changes is particularly sparse. We have previously expanded the list of deleterious SSU mt-rRNA mutations by identifying highly disruptive base changes capable of blocking the activity of the mitoribosomal SSU. To do this, we used a new methodology named heterologous inferential analysis (HIA). The recent arrival of near-atomic-resolution structures of the human mitoribosomal LSU, has enhanced the power of our approach by permitting the analysis of the corresponding sites of mutation within their natural structural context. Here, we have used these tools to determine whether LSU mt-rRNA mutations found in the context of human disease and/or ageing could disrupt the function of the mitoribosomal LSU. Our results clearly show that, much like the for SSU mt-rRNA, LSU mt-rRNAs mutations capable of compromising the function of the mitoribosomal LSU are indeed present in clinical samples. Thus, our work constitutes an important contribution to an emerging view of the mitoribosome as an important element in human health.

## Highlights

• Identification of pathogenic mutations of mitochondrial rRNAs is problematic.
• We analysed 64 rare 16S rRNA mutations obtained from clinical samples.
• The mutations underwent *h*eterologous *i*nferential *a*nalysis (HIA).
• We show that highly disruptive 16S rRNA mutations are present in clinical samples.

## 1 Introduction

Mitochondria are the cellular powerhouses and contain a small circular chromosome (mtDNA). Human mtDNA codes for 13 essential polypeptide components of the mitochondrial oxidative phosphorylation chain, which generates most of the cells' ATP. More than 250 pathogenic mutations and large-scale re-arrangements of the mitochondrial chromosome have been identified, as causative of a heterogeneous group of diseases. Most pathogenic mutations are present as heteroplasmic variants, meaning that both wild-type and mutated mtDNA co-exist within the cells of patients. The precise threshold level of mutant mtDNA required for a major biochemical defect within the cell depends on the mutation, and shows variation with tissue type and between individual patients. This results in a complex and heterogeneous pattern of disease presentation (Tuppen et al., 1797). Indeed the penetrance of mtDNA mutations is highly variable, and known to be influenced by a number of factors. It has been estimated that the minimal prevalence of mtDNA disease in adults is 1 in 4300 in the UK (Gorman et al., 2015). As such, mtDNA mutations must be considered an important cause of inherited disease. In addition, the high mutation rate of mtDNA within cells and its continuous turnover independent of cell cycle, results in the accumulation of somatic mutations with time (Wallace, 2010, Elson et al., 2001). For this reason, the detection of somatic mtDNA mutations in aged and cancerous tissues is amply documented in the literature.

In addition to 13 essential polypeptides, mtDNA codes for a number of components of the machinery required to synthesise these polypeptides, namely 2 ribosomal RNAs (rRNAs) and 22 transfer RNAs (tRNAs) (Scheffler, 1999). Mt-rRNA genes encode the RNA component of mitochondrial ribosomes or mitoribosomes. The mammalian mitoribosome consists of a 28S *s*mall *su*bunit (mt-SSU), where genetic decoding occurs, and a 39S *l*arge *su*bunit (mt-LSU), which holds the peptidyl transferase activity (Koc et al., 2010). Given the central role of the mitoribosome in mitochondrial gene expression, it is not surprising that some mt-rRNA mutations have emerged as causative of mitochondrial disease. Our understanding of the role, diagnosis and transmission of mutations in the mtDNA protein encoding genes and mt-tRNA genes has improved greatly over the last 10 years, at least in European populations (Mitchell et al., 2006, Yarham et al., 2011, Stewart et al., 2008, Elson et al., 2009). However, much less is known about disease causing mutations in the mt-rRNA genes than in any of the other genes of mtDNA. Thus, efforts to expand our knowledge of the importance of mt-rRNA variation in disease may be considered one of the last frontiers in the understanding of the role of mtDNA mutations in human disease.

The intractability of the mitochondrial translation apparatus, together with the lack of methods for the manipulation of mtDNA makes it impossible to biochemically and genetically test the effect of these mutations directly. As such, it is not surprising that to date, only two mt-rRNA mutations exist for which direct biochemical evidence has clearly established a role in the onset mtDNA disease (Bindu and Reddy, 2008, Smith et al., 2014). In an attempt to identify more mt-rRNA mutations capable of interfering with mitoribosomal function, we have described a method named HIA, which we used to assess the disruptive potential of mt-rRNA variations in the mt-small ribosomal subunit (mt-SSU) (Smith et al., 2014, Elson et al., 2015). The method is based on the striking evolutionary conservation of the overall fold of rRNAs, which attests to both the common ancestry of these molecules and their pivotal biological role (Cannone et al., 2002). HIA combines conservational information with functional and structural data obtained from heterologous ribosomal sources, and can be used to make predictions of the disruptive power of mt-rRNA variants (Smith et al., 2014). We have successfully used HIA to reveal the pathogenic character of certain mt-rRNA mutations in the mt-SSU for which the available genetic and pathological evidence was unambiguous (Smith et al., 2014). Notably, a structure-based method similar to ours have recently been applied to predict the disruptive potential of mtDNA mutations within protein-coding genes (Lloyd and McGeehan, 2013).

Recent advances in cryo-EM technology have allowed the achievement of medium- and near-atomic-resolution structures of mitochondrial ribosomal particles, including a 3.4 Å-resolution structure of the *Homo sapiens l*arge *su*bunit (mt-LSU) (Brown et al., 2014). The availability of this structure is a major step towards achieving a better understanding of the role of mt-rRNA mutations in human disease. In addition to facilitating direct location of sites of variation on the correct structure, it has allowed the advancement of our HIA analysis to assess the functional and/or structural role of such sites. Here, we use these tools to analyse the effects of LSU mt-rRNA mutations on the function of the large mitoribosomal subunit.

## 2 Methods

A detailed description of the HIA methodology has been recently published (Smith et al., 2014, Elson et al., 2015). Aspects of particular importance to this work are described below.

### 2.1 Sources of mutational data

The following sources of mutational data were used in this study: (a) scientific literature, (b) MITOMAP list of unpublished variants (Ruiz-Pesini et al., 2007), and (c) our own unpublished material. As for our previous work on the small mitoribosomal rRNA (Smith et al., 2014), the criterion required for mt-rRNA mutations to be included in this study was their identification in human subjects, with either a suspected mitochondrial disease or somatic mtDNA mutations that had undergone clonal expansions in cancer patients, or COX-deficient cells located in aged tissues. In the case of studies involving tumourigenic tissue, the existence of non-cancerous control tissue from the same patient was a requirement. Similarly, the existence of COX-proficient surrounding tissue was a requirement in the case of COX-deficient samples of aged individuals. This allowed the variant to be identified as a somatic mutation that had undergone clonal expansion.

### 2.2 Analysis of GenBank mitochondrial genome data

We have previously described our strategy to filter out mutations with appearances in samples obtained from normal individuals (Smith et al., 2014, Elson et al., 2015). As of the end of 2013, there existed 21,591 human mtDNA sequences in GenBank (query = ‘Homo[Organism] + AND + gene_in_mitochondrion[PROP] + AND + 14,000:19,000[SLEN] + NOT + pseudogene[All Fields]’ (Pereira et al., 2009)). All sequences were downloaded with Entrez Programming Utilities and our own PERL scripts (Sayers, 2010). The data was downloaded in FASTA format and sequentially aligned to the Cambridge Reference (NC_012920.1) (Andrews et al., 1999) by means of the MAFFT package (Katoh et al., 2002), which provided a quick algorithm for massive sequence alignment. The generated alignments were then analysed by means of a PERL script that recorded the total number of appearances for every variation. For variations with 15 or less appearances in the combined dataset, including the data from both the GenBank and Phylotree databases, the possibility that the individual sequences could be linked to mitochondrial disease was investigated, by inspecting the related publication sources. In all cases it was observed that no new patient sequences, other than the ones previously found in the literature, were present within GenBank.

In addition to this strategy, here we have also used the tools available within the MITOMAP database (Ruiz-Pesini et al., 2007). Only mutations passing these filters, were analysed in this work (Supplementary Table 1).

### 2.3 Structural inspection

The improved version of the HIA method used here includes the following changes. The availability of a 3.4 Å-resolution structure of the *H. sapiens* mt-LSU (Brown et al., 2014), has prompted us to use this structure to directly place the sites of variation under analysis (RCSB accession code: 3J7Y). Direct structural analysis on structure 3J7Y was complemented with comparisons to superimposed structures representative of all three kingdoms of life and mitochondria. In the bacterial case, the availability of structures in different steps of the translation cycle was also exploited. The structures used in this analysis are described in Supplementary Table 2. The quality of 3J7Y's RNA density was visually evaluated prior to HIA analysis.

Tertiary and quaternary interactions are visually assigned with UCSF Chimera on superimposed high-resolution structures of LSUs belonging to bacterial (*Thermus thermophilus* and *Escherichia coli*), eukaryotic, archaeal, and mitochondrial (including human and yeast mitochondria) phylogenetic domains (Supplementary Table 2). Superimpositions were performed in Chimera UCSF, as described (Elson et al., 2015, Pettersen et al., 2004).

To show the positions of A- and P-site tRNAs in Fig. 2, the following superimposition strategy was followed. RCSB ID 2WDK, containing the 3.5 Å structure of the *T. thermophilus* SSU plus all three tRNAs, was superimposed onto 2J00, a higher resolution structure from the same organism (2.8 Å, Supplementary Table 2) (Voorhees et al., 2009, Selmer et al., 2006). The RMSD between 1476 atom pairs was 0.700 Å in this case. On a second step, RCSB ID 3J7Y, carrying the 3.4 Å human mitoribosomal LSU structure was superimposed onto 2J01, the LSU of 2J00 (Supplementary Table 2), with an RMSD of 0955 Å between 410 atom pairs (Brown et al., 2014, Selmer et al., 2006). For the mitochondrial SSU, the 7.0 Å *Bos taurus* structure (RCSB ID: 3J6V) was superimposed onto 2J00 (Kaushal et al., 2014), with an RMSD of 1.452 Å between 83 atom pairs.

### 2.4 Disruptive power assessment

As highlighted in our previous studies (Smith et al., 2014), the disruptive potential was estimated as follows: N = ‘certainly not disruptive’, supportive direct heterologous mutagenesis data in favour of this assignment exists for the tested residue or its base-pairing partner, U = ‘unlikely disruptive’, no direct heterologous mutagenesis data exist but enough indirect data exist in favour of this conclusion; NEE = ‘not enough evidence’, no direct or indirect evidence argues against a potential disruptive power; L = ‘likely disruptive’, no direct heterologous mutagenesis data exist but enough indirect data exist in favour of this conclusion and E = ‘expectedly disruptive’, supportive direct heterologous mutagenesis data exist for the tested residue or its base-pairing partner. Two additional categories were used to classify the mitochondrial mutations: und = ‘undetermined’, no heterologous data exist to evaluate the disruptive potential or the existing structural differences are too large to allow the extrapolation of conclusions made in the heterologous case and P='proven', direct biochemical evidence exists to support a disruptive role.

### 3 Nomenclature

Conserved interactions are indicated in the main text as a ^C^ followed by the corresponding ^beamM^, for observed interactions in the bacterial, eukaryotic, archaeal, mammalian mitochondrial, and yeast mitochondrial ribosomes. In the absence of the corresponding symbol, a plus sign (+) indicates that all the partners for a potential interaction are present in a particular organism, but the interaction has not been modelled in the published structure, a minus (−) sign denotes that one of the partners in an interaction is absent in a particular organism, and a question mark (?) indicates that the interaction is not observed in a particular organism despite the fact that potential partners for it are present. For the description of the higher-order structure of the mt-LSU, mitochondrial numbering (regular font) refers to *H. sapiens*. *E. coli* numbering is the accepted standard for rRNA, therefore it is used here to denote bacterial rRNA residues (italicised). Conservation indexes, **C**_**v**_s, calculated as described by Cannone et al. (2002), are also provided. All human sites of variation tested here, as well as their heterologous equivalents will be underlined. Atom-to-atom distances below 3 Å are reported.

## 4 Results

**Fig. 1:**
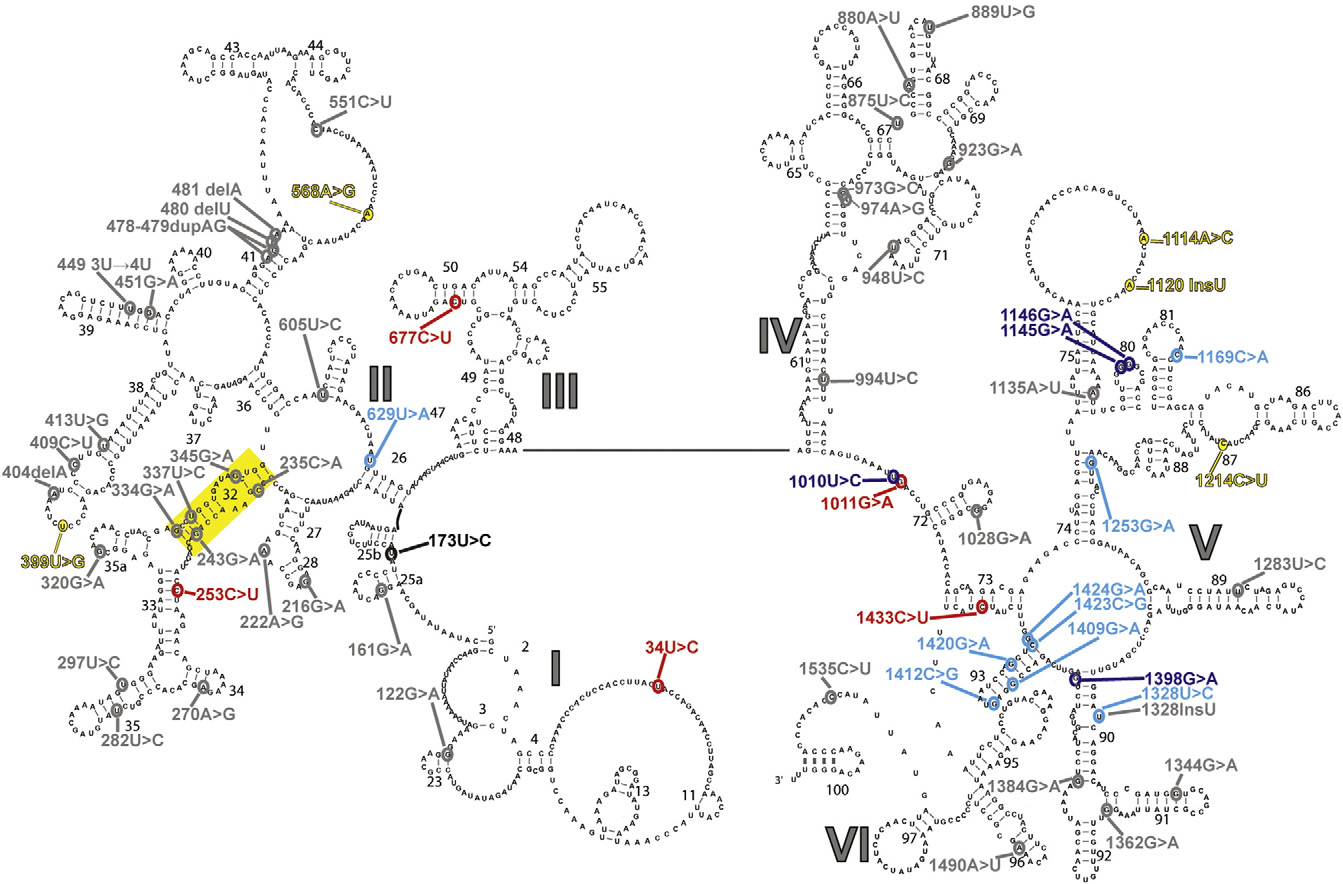
Secondary structure of the human LSU mt-rRNA. Modified from Brown et al. (2014). rRNA domains are indicated by large Roman numerals. Helix 32 is indicated with solid yellow box. Sites of variation are colour coded as follows: Red, “unlikely”; yellow, “undetermined”; grey, “NEE”; cyan, “likely”; blue, “expectedly”; black, “proven”.

We have analysed 145 potentially disruptive mutations mapping to the human 16S mt-rRNA. Among these mutations, we identified 64 variants with no appearances in GenBank, other than the ones originating from the sources reporting them as potentially pathogenic (Supplementary Table 1). Fig. 1 shows the sites of these mutations displayed on the secondary structure of the human mt-rRNA. Improved HIA analysis resulted in: 6 variants whose disruptive potential on the function of mitoribosomes was considered to be “unlikely disruptive”, and 5 which cannot be placed on the high-resolution human cryo-EM structure of the mt-LSU (undetermined) (Brown et al., 2014). An additional 36 variants were regarded as not having enough evidence “NEE”, 12 variants were deemed “likely disruptive”, 4 were considered “expectedly disruptive”, and 1 mutation was confirmed as “proven” (Table 1). What follows is an analysis of the relevant conclusions from the point of view of mitochondrial function and human disease, for mutations within the “likely”, “expectedly”, and “proven” categories.

## 5 Domain I mutations

**Fig. 2:**
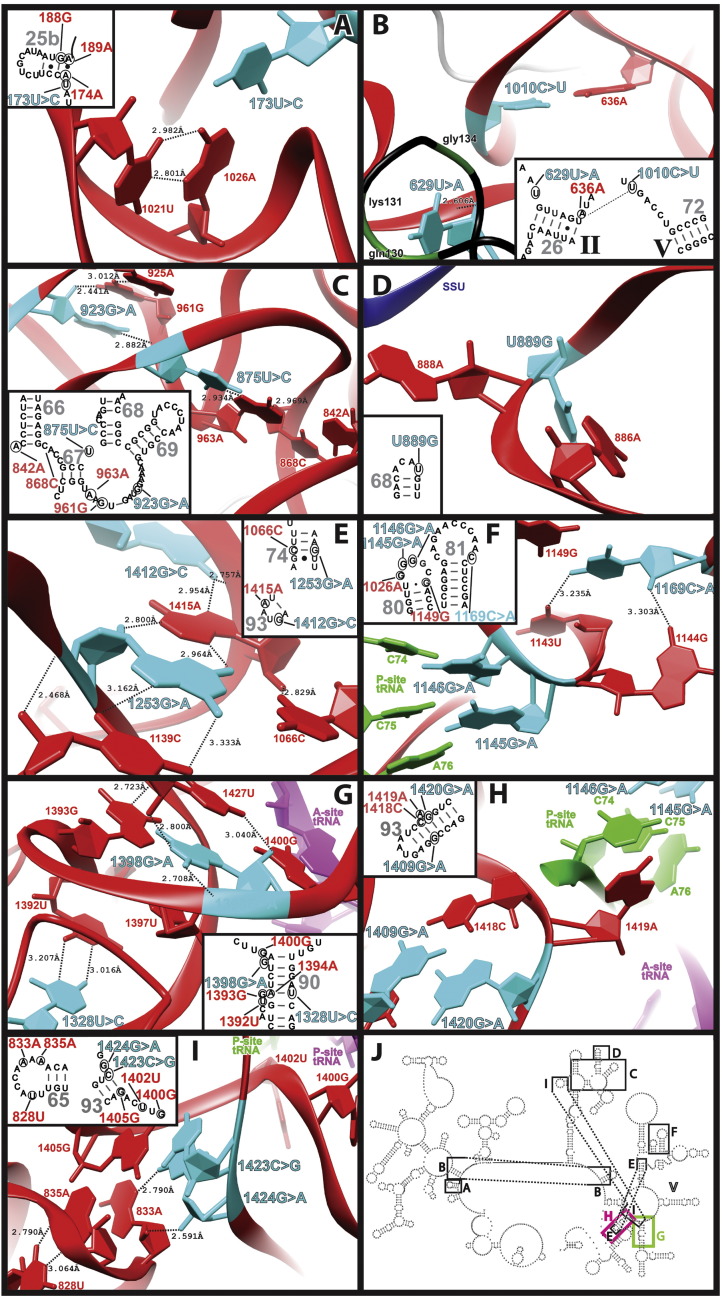
Structural analysis of variants with high disruptive potential. A) 173U > C (m.1843T > C). B) 629U > A (m.2299T > A) and 1010U > C (m.2680T > C). C) 875U > C (m.2545T > C) and 923G (m.2593G). D) 889U > G (m.2559T > G). E) 1253G > A (m.2923G > A) and 1412G > C (m.3082G > C). F) 1145G > A (m.2815G > A), 1146G > A (m.2816G > A), and 1169C > A (m.2839C > A). G) 1328U > C (m.2998T > C) and 1398G > A (m.3068G > A). H) 1420G > A (m.3090G > A) and 1409G > A (m.3079G > A). I) 1423C > G (m.3093C > G) and 1424G > A (m.3094G > A). J) Secondary-structure diagram with explanatory boxes indicating the location of the sites of mutation shown in panels A through I. Boxes G and H are coloured in magenta and light green to simplify the visualization of the figure. Variant sites in A–I are shown in aquamarine, associated sites in red. Ligands and atomic distances are indicated. tRNA structures in F, G, H, and I and SSU structure in D were superimposed as described in Methods. Insets correspond to the areas boxed in panel J. Helices are indicated in insets with large grey numbers. rRNA domains are indicated in insets with Roman numerals. Variant and associated sites in insets are colour coded as on main panel.

A potentially pathogenic mutation was identified in a respiratory chain-deficient cybrid cell line by Seibel et al. at position 173U (m.1843T > C) (Seibel et al., 2008). Residue 173U lies between helices H25a and H25b and is part of a triple base interaction involving residues of helix H72 (Fig. 1, Fig. 2A). This interaction, which contributes to bringing together domains I and V of the LSU rRNA, is established via a Watson:Crick base pair between 173U and 1026A (m.2696A), and a reverse Hoogsteen between 1026A and 1021U (m.2691U) of H72 (bacterial: *U562**:A2033*·*U2028*; **C**_**v(*****U562*****)**_ = 0.992, **C**_**v(*A2033*)**_ = 1.292, **C**_**v(*U2028*)**_ = 1.197)^CbeamM^. The 173U > C transition is likely to disrupt the triple base interaction even if a C·A^+^ wobble could be formed between the mutated 173C and 1026A. This is because the protonation of the N1 of 1026A would disrupt the reverse Hoogsteen configuration (Pan et al., 1998, Saenger, 1984). The loss of base pairing interactions in the neighbourhood of this triple base interaction was demonstrated to be highly detrimental to ribosomal function by mutagenesis in bacteria (specifically, residues *2025* and *2026*) (O'Connor, 2007, Thompson et al., 2001a). Notably, mutations at the adjacent *G2032* of the bacterial ribosome are known to cause resistance to the oxazolidinone antibiotic linezolid (Kloss et al., 2000). Interestingly, functional analysis of cybrid cell lines carrying this mutation in addition to the NEE variant 270A ≥ G (m.1940A > G), showed a dramatic reduction in the amount of mitochondrially encoded proteins (Seibel et al., 2008). It is likely that the observed deleterious effects can be mostly attributed to the 173U > C (m.1843T > C) mutation, as the NEE variant 270A > G (m.1940A > G) is not expected to cause major structural defects, (Table 1). Given this evidence, the 173U ≥ C (m.1843T > C) mutation is classified as a “proven disruptive” mutation.

## 6 Peptide exit tunnel (ET)

Two interesting disruptive mutations map to the PET. This structural element spans the distance from the peptidyl transferase centre (PTC) to the peptide exit site on the opposite end of the large subunit (Pechmann et al., 2013). The folding of nascent peptide chains starts within the PET, where they can even form helices (Pechmann et al., 2013). In mammalian mitochondria, the tunnel appears to be adapted for the translation of hydrophobic membrane proteins (Brown et al., 2014).

A U > C heteroplasmic variant was identified at position 1010 (m.2680) in a breast cancer tumour sample (Gochhait et al., 2008). Note that the assignment of a C > U at this position in the original report was a typographical error (personal communication from Dr. Bamezai). In the human cryo-EM structure, 1010U forms a Watson–Crick base pair with 636A (bacterial: *A1262:**U2017*; **C**_**v(*A1262*)**_ = 0.525 and **C**_**v(*****U2017*****)**_ = 0.638), a residue flanking one of the ends of helix H26 on the wall of the peptide exit tunnel (Fig. 1, Fig. 2B). Base changes disrupting the bacterial equivalent of this base pair have been shown to be highly detrimental for ribosome function (Aagaard and Douthwaite, 1994). While it is possible that the U > C base change at position 1010 (m.2680) might result in an A^+^·C wobble, the bacterial studies showed that the wobble configuration at this site was not sufficient to fully restore wild type activity (Aagaard and Douthwaite, 1994). As such, the evidence prompts us to regard the U > C heteroplasmic variation at m.2680 as “expectedly disruptive”.

The opposite end of helix H26 in the human mitoribosome also harbours the potentially pathogenic mutation 629U > A (m.2299T > A). This variant was identified in a sample from a patient with colorectal cancer by Polyak et al. (1998). In the human mitochondrial structure 629U (bacterial *U1255*; **C**_**v(*****U1255*****)**_ = **1.848**) is stacked upon a loop of protein MRPL4 whose primary sequence is highly conserved between bacteria and mitochondria (Fig. 1, Fig. 2B; an alignment between MRPL4 and the bacterial L4 is provided as Supplementary material). The loop forms part of the lining of the peptide exit tunnel, and in bacteria it is known to harbour mutations that confer erythromycin-resistance (Zaman et al., 2007). A contact between the ribose of 629U and the phosphate oxygen of 189A of helix H25b^Cbeam^ (bacterial: *A578*; **C**_**v(*A578*)**_ = 0.884) is also observed (Fig. 2B). The high conservation of 629U, together with its involvement in long-range interactions suggests an important role for this residue. In agreement with this hypothesis, is the observation in bacteria that mutations of neighbouring residues of protein L4 produce slow growing phenotypes (Zaman et al., 2007). Thus, we regard 629U > A (m.2299T > A) as a “likely disruptive” mutation.

## 7 Domain IV bridges and associated regions

Domain IV of the bacterial LSU is involved in the formation of several inter-subunit bridges that link the two ribosomal subunits. This region of the ribosome has been implicated in numerous ribosomal functions including subunit association, tRNA binding, initiation, translocation, translational accuracy, peptidyl transferase reaction, and ribosome recycling (Ali et al., 2006).

### 7.1 Bridges B2b and B2c

The importance of these bridges in subunit association has been clearly demonstrated (Ghosh and Joseph, 2005, Rackham et al., 2006). On the LSU, bridges B2b and B2c involve residues of helices H67, H69, and H71, and of helix H67 respectively (Fig. 1) (Yusupov et al., 2001). We will examine three mutations that lie in close proximity of these bridges.

Linking helices H67 and H68 is the almost invariant residue 875U (bacterial: *U1834*; **C**_**v(*****U1834*****)**_ = 1.913) (Figs. 1 and 2C), where a C > U mutation has been identified in a sample from a patient with prostate cancer (Kloss-Brandstatter et al., 2010). Position 875U lies in the neighbourhood of another highly conserved site of mutation, namely 923G (m.2593G) (*H. sapiens*: 923G; bacterial: *G1930*; **C**_**v(*****G1930*****)**_ = 1.963) (Figs. 1 and 2C), where a G > A base change has been identified in a COX-deficient gastric unit (McDonald et al., 2008).

The high degree of conservation of positions 875U (m.2545T) and 923G (m.2593G) is likely to be explained by their probable role in local structure stabilization, in particular the clamping of the sharp bend between residues 961 and 966 (grey face in *H sapiens*, and *E. coli* figure) (bacterial: *1968* and *1973*; **C**_**v(average)**_ = 1.683) (Fig. 2C). The N3 of 875U is within hydrogen-bond distance of the N7 of 963A^CbeamM^ (bacterial: *U1834*·*A1970*; **C**_**v(*****U1834*****)**_ = 1.913, **C**_**v(*A1970*)**_ = 1.881) at the very apex of the sharp bend. This interaction is continued via a contact between the N3 of 963A and the 2'OH of 868C (bacterial: *C1827*; ***C***_***v*(*C1827*)**_ = 1.383)^CbeamM^ that connects the bend to helix H66. This continuous hydrogen-bonding network reaches even further due to the formation of a C·A^+^ wobble between 868C and 842A (bacterial: *G1792*; **C**_**v(*G1792*)**_ = 1.116).

The case of 923G is similar to that of 875U. The Watson–Crick face of 923 is modelled to be within less than 3 Å of the RNA backbone at position 962A^CbeamM^ (bacterial: *A1969*; **C**_**v(*A1969*)**_ = 1.952) (Fig. 2C), while its ribose 2'OH makes a contact with 961's base^CbeamM^ of the highly conserved 925A·961G sheared base pair (*H. sapiens*: 925A·961G; bacterial: *A1932*·*G1968*, **C**_**v(*A1932*)**_ = 1.947; C_v(*G1968*)_ = 1.852)^CbeamM^.

Regarding the possible effects of the 875U > C (m.2545T > C) and 923G > A (m.2593G > A) mutants, the mitochondrial structure indicates that the U > C substitution at position 875 would result in the loss of the N3–N7 hydrogen bond with 963A. Similarly, the G > A base change at position 923 would also result in the loss of one hydrogen bond. Given the high conservation of the residues in this region, their involvement in maintenance of local structure, and the proximity to bridges B2b and B2c; it appears logical to propose that such losses of hydrogen-bonding potential in this region should be deleterious. Mutagenesis of some adjacent bridge residues that has been performed in bacteria corroborates this idea (Liiv and O'Connor, 2006). For example, mutation of residues neighbouring B2b resulted in biochemical and growth defects, including some recessive lethal phenotypes. However, it was also shown that B2c bridge mutants that maintained base pairing across helix H67 result in relatively minor growth and subunit association defects, whereas mutations that did not maintain base pairing across helix H67 led to phenotypes of greater severity (Liiv and O'Connor, 2006). Furthermore, lack of methylation at position *G1835* in bacteria (*H. sapiens*: 876A; **C**_**v(*G1835*)**_ = 1.861) has been shown to cause important growth defects under osmotic and oxidative stress, situations that might be relevant to mitochondrial translation (Osterman et al., 2011). Finally, deleterious mutations have also been found on the SSU side of the bridge in bacteria (Ghosh and Joseph, 2005, Rackham et al., 2006, Kim et al., 2007, Belanger et al., 2004) and we have previously identified two “likely disruptive” variants in the 12S mt-rRNA component of bridge mt-B2c (Smith et al., 2014). In summary, the data presented here is consistent with a “likely disruptive” potential for the two LSU mutations located on bridge mt-B2c, namely 875U > C (m.2545T > C) and the 923G > A (m.2593G > A). In the case of the 875U > C (m.2545T > C), a second LSU somatic mutation has been observed in the same sample (Supplementary Table 1), mapping to position 337U (m.2007T) within the PET (Kloss-Brandstatter et al., 2010). As the two sites of mutation are far apart in the LSU structure and we assigned a “NEE” score to 337U (m.2007T) (Table 1), the possibility of a synergistic, disruptive effect of the two base changes cannot be ruled out.

### 7.2 Bridge B7a

Superposition of the recent cryo-EM structures of the bacterial and mammalian mitoribosomal subunits shows that the surface area involved in bridge B7a is smaller in the mammalian mitoribosome than in its bacterial counterpart, due to the shorter length of helix H68 in the former (Brown et al., 2014, Kaushal et al., 2014, Greber et al., 2014).

The 889U > G (m.2559T > G) variant was identified in a COX-deficient colonocyte at levels of 50% heteroplasmy (Greaves et al., 2010). In the human mitochondrial structure 889U forms a Watson–Crick base pair with 886A (m.2556A) within H68's capping loop (Fig. 1, Fig. 2D). The available structural data places position 889A at the very centre of the mitochondrial bridge, i.e. a distance of less than 4 Å can be measured between the phosphate oxygen of 889A and the RNA backbone of the superimposed *B. taurus* 7.0 Å cryo-EM structure (Fig. 2D) (Brown et al., 2014, Kaushal et al., 2014).

While caution should be used when interpreting the aforementioned structural details, given the low resolution of the *B. taurus* structure, the location of the 889U > G (m.2559T > G) prompts us to consider its disruptive potential as “likely disruptive”.

### 7.3 Ribosomal peptidyl transferase centre (PTC)

The PTC is one of the essential functional centres of the ribosome, as it is responsible for peptide bond formation. In addition, the PTC defines the entrance to the exit tunnel used by nascent peptides on their way out of the ribosome. Given its central role it is widely believed that the PTC is one of the oldest polymeric elements of biological systems. It is composed of helices H74, H80, H89, H90, and H91–H93 of domain V (Fig. 1) (Petrov et al., 2014). Not surprisingly, this is the region of the LSU that contains most of the mutations with the highest disruptive potential.

The 1253G > A (m.2923G > A) variant was observed as a homoplasmic variant in a sample of tissue from a prostate cancer patient (Jeronimo et al., 2001). The canonical 1253G:1066C base pair, mapping to helix H74, forms tertiary interactions via its major and minor grooves with positions 1139C and the nearly invariant 1415A of helix H93 (A-minor motif), respectively (bacterial: (*A2598*·{*C2073:[**G2436*})·*U2245]*; **C**_**v(*A2598*)**_ = **1.913**, **C**_**v(*C2073*)**_ = **1.694**, **C**_**v(*****G2436*****)**_ = **1.726**, **C**_**v(*U2245*)**_ = **1.162**)^CbeamM^ (Fig. 2E). This interaction is extended via a double hydrogen bond interaction between 1415A and the structurally adjacent 1412G of helix H93. A heteroplasmic G > C base change has been detected at position 1412G (m.3082G) (bacterial: *G2595*; **C**_**v**(*G2595*)_ = 1.957) in a sample from an oesophageal cancer (Liu et al., 2012). The importance of these interactions is clear, as they allow the integration of several RNA elements needed to build the peptidyl transferase region. First, the single stranded stretch joining helices H75 and H80 interacts with the major groove of H74 via the aforementioned contacts involving residue 1139C ^CbeamM^. On the minor groove side of helix H74 contacts are made to the terminal loop of helix H93 that involves residues 1412G and 1415A^CbeamM^.

Notably, helices H80 and H93 carry key residues involved in the correct positioning of the A- and P-site tRNAs at the PTC; as such, it would be expected that disruption of local structure in this region could seriously affect ribosomal function. Supporting this view, mutations at the neighbouring 1255U (*bacterial: U2438*; **C**_**v**(*U2438*)_ = 1.847) have been generated in *Halobacterium halobium*, showing a degree of disruption that was in agreement with the predicted level of local structure distortion (Leviev et al., 1994). Taking all these data together, it appears reasonable to propose that the 1253G > A (m.2923G > A) and 1412G > C (m.3082G > C) mutations (Jeronimo et al., 2001, Liu et al., 2012), both resulting in loss of hydrogen bonding potential, should yield phenotypes that disrupt ribosome function. Given this, we regard both mitochondrial variants as “likely disruptive”. Additionally, the universal conservation 1412G was also considered an important factor in the assignment as “likely disruptive”, as no instances of G > A substitution at this position exist, as determined by Cannone et al. (2002).

#### 7.3.1 P-loop

Two interesting mutations have been mapped to helix H80 whose capping loop is known as the P-loop. The 1145G > A (m.2815G > A) variant was found independently in a COX-deficient colonic crypt (60% heteroplasmy) (Fig. 1) (Taylor et al., 2003) and as a homoplasmic base change in a COX-deficient urothelium patch (Gaisa et al., 2011). In addition, a G > A variation was detected as homoplasmic at the adjacent residue, 1146G (m.2816G), again in a COX-deficient colonocyte (Greaves et al., 2010). Mutagenesis studies performed in bacteria have shown that base changes at the equivalent residues, *G2251* (**C**_**v**(*G2251*)_ = 1.987) and *G2252* (**C**_**v***G2252*)_ = 1.912), and at the adjacent *G2253* (**C**_**v**(*G2253*)_ = 1.781) are dominant lethal (Samaha et al., 1995, Lieberman and Dahlberg, 1994, Green et al., 1997). The reason for this dramatic phenotype is related to the involvement of these residues in the peptidyl transferase reaction. Specifically, Watson–Crick interaction between *G2252* and *G2251* in the so-called P-loop of H80, and positions C74 and C75 on the acceptor end of P-site tRNA are essential for proper substrate positioning during peptidyl transfer (Fig. 2F) (Selmer et al., 2006, Samaha et al., 1995, Green et al., 1997, Nissen et al., 2000, Feinberg and Joseph, 2006). Since it is expected that mutations at the mitochondrial equivalents should result in similar phenotypes, the 1145G > A (m.2815G > A) and 1146G > A (m.2816G > A) variants are regarded as “expectedly disruptive” (Taylor et al., 2003, Gaisa et al., 2011).

Yet another mutation mapping to the neighbourhood of the P-loop is the 1169C > A (m.2839C > A) variant, which has been detected as a heteroplasmic variant in a patient with Dupuytren's disease (Fig. 1) (Bayat et al., 2005). Position 1169C canonically base pairs with 1149G (bacterial: *G2255:**C2275*; **C**_**v(*G2255*)**_ = 1.683; **C**_**v**(*G2275*)_ = 1.650) bringing together the P-loop and helix H81 (Fig. 2F). In summary, the location of the 1169C > A (m.2839C > A) variant in the close neighbourhood of the functionally important P loop, together with the structurally disruptive character of the base change makes this mutation a candidate for a “likely disruptive” mutation.

#### 7.3.2 H90

The 1328U > C (m.2998T > C) variant has been identified as a heteroplasmic variant in a patient with breast cancer (Fendt et al., 2011). 1328U is part of a non-canonical pyrimidine–pyrimidine base pair interaction involving residue 1392U within helix H90^Cbeam^ (bacterial: *U2511*·*C2575*; **C**_**v(*****U2511*****)**_ = **1.930**, **C**_**v(*C2575*)**_ = **1.135**) (Fig. 1, Fig. 2G). The adjacent residue, 1393G (bacterial: *G2576*; **C**_**v(*G2576*)**_ = **1.804**) is flipped out of the helix and modelled in the syn conformation^CbeamM^. This conformation positions 1393G's N1 within hydrogen bonding distance of the RNA backbone at position 1323U^Cbeam?^, an almost universally conserved residue (bacterial: *U2506*; **C**_**v(*U2506*)**_ = **1.976**) that in bacteria has been shown to be directly involved in the binding of the A-site amino acid at the PTC (Fig. 2G) (Voorhees et al., 2009). Given this location, it is expected that mutations resulting in the disruption of local structure could seriously affect peptidyl transferase and/or tunnel activities. Indeed, mutations at the bacterial equivalent of position 1393G resulted in almost 3-fold increase in growth rate and conferred resistance to the antibiotic linezolid (Long et al., 2010). In light of these data, it appears logical to suggest that the 1328U > C (m.2998T > C) variant identified by Fendt et al. (2011) should be regarded as a “likely disruptive” mutation with potential to cause linezolid hyper susceptibility. Interestingly, a homoplasmic U insertion has been detected at the same position in a patient with lung cancer (Lorenc et al., 2003). Regarding this insertion, it is not clear whether it might cause enough local distortion to result in peptidyl transferase defects and/or linezolid hyper susceptibility. As a result, the insertion variant is considered “NEE”.

We have identified a homoplasmic G > A mutation at the nearly invariant position 1398G > A (m.3068G > A) in a COX deficient tumour from a patient with a tubulovillus adenoma of the rectum (bacterial: *G2581*; **C**_**v(*G2581*)**_ = **1.905**). 1398G maps to helix H90, where it is modelled in the syn conformation^CbeamM^ (Fig. 1, Fig. 2G). This conformation is stabilized by intra-chain hydrogen bonds between 1398's base and the RNA backbone^CbeamM^ and by its stacking between the universally conserved 1397U (m.3067U)^CbeamM^ (bacterial: *U2580*; **C**_**v(*U2580*)**_ = **2.000**) and 1427U (m.3097U) (bacterial: *C2610*; **C**_***v(C2610)***_ = **0.991**)^Cbeam −^ (Fig. 2G). Particularly important is the role of neighbouring position 1400G (bacterial: *G2583*; ***C***_***v(G2583)***_ *=* ***1.969***), which in bacteria stabilizes the A-site tRNA in the PTC by forming a Class I A-minor interaction with position A76 of this tRNA, a residue responsible for orienting the incoming amino acid for nucleophilic attack on the peptidyl ester (Voorhees et al., 2009). In agreement with these observations, mutagenesis experiments have shown that mutations of residues in this region are highly deleterious (Spahn et al., 1996a, Spahn et al., 1996b, Porse et al., 1996). In particular, the G to A mutation at position *2581* conferred a dominant lethal phenotype in *E. coli* (Spahn et al., 1996b). Given the COX-deficiency detected in association with the 1398G > A (m.3068G > A) base change, together with its homoplasmic status, we are prompted to view it as an “expectedly” disruptive mutation. Much like the P loop mutations, the COX-deficiency observed in the tumour, makes an important case for the consideration of 1398G > A (m.3068G > A) as a proven mutation.

#### 7.3.3 H93

We have found a few variants in helix H93 that could affect the correct positioning of tRNAs in the PTC. For example, the 1409G > A (m.3079G > A) variant was independently identified in samples from two patients with squamous cell carcinomas (Zhou et al., 2007, Mithani et al., 2007). This residue base pairs with position 1418C within helix H93 (bacterial *G2592**:C2601*; **C**_**v(*****G2592*****)**_ = **1.923**, **C**_**v(*C2601*)**_ = **1.779**) (Fig. 1, Fig. 2H). Immediately adjacent to this base pair lies another site of variation, namely 1420G (m.3090G) where a G > A base change has been identified in a 20-year-old woman with severe myopathy (Coulbault et al., 2007). The variation was almost homoplasmic in skeletal muscle fibres, seen at 50% heteroplasmy in urine; while being absent in fibroblast, jugal cells, hair follicles, and blood cells (Coulbault et al., 2007). In the human structure, 1420G base pairs with position 1408C (bacterial: *C2591:**G2603*; **C**_**v(*C2591*)**_ = **1.955**, **C**_**v(*****G2603*****)**_ = **1.967**) (Fig. 2H). Next to 1418C is the almost invariant 1419A (bacterial: *A2602*; **C**_**v(*A2602*)**_ = **1.939**), which in bacteria is placed between the acceptor ends of P- and A-site tRNAs (Voorhees et al., 2009, Selmer et al., 2006). In agreement with its location, mutations at this residue are dominant lethal, arguing for an important role of the residue in the chemical reactions occurring at the PTC (Polacek et al., 2003, Youngman et al., 2004). In addition to stabilizing the structure around position 1419A, both 1409G and 1420G mediate contacts between helix H93 and helices H70/H71, thus linking the peptidyl transferase centre to domain IV of the LSU rRNA. Given the crucial role of H93 in the chemistry of the PTC, it is expected that even a small distortion of local structure in this region could have catastrophic effects on this chemistry. In the case of 1409G > A (m.3079G > A) and 1420G > A (m.3090G > A), even the potential formation of wobble base pairs as a result of the G > A base changes might impose sufficient distortion to result in defective ribosomes. As a result, the two variations are considered “likely disruptive”.

The 1423C ≥ G (m.3093C > G) and 1424G ≥ A (m.3094G > A) mutations map to the proximal end of helix H93 (Fig. 1, Fig. 2I) (Taylor et al., 2003, Hsieh et al., 2001). 1423C ≥ G (m.3093C > G) was identified in a patient with MELAS syndrome complicated with diabetes mellitus, hyperthyroidism and cardiomyopathy (Hsieh et al., 2001), and the 1424G ≥ A (m.3094G > A) variant was detected at level of 40% mtDNA heteroplasmy in a COX-deficient colonic crypt (Taylor et al., 2003).

In the case of 1423C > G (m.3093C > G), the mutation was present at 51% heteroplasmy in skin fibroblasts of the proband, but at much lower levels in other tissues and absent in blood. Interestingly, the proband also carried the inherited MELAS mutation 3243A > G (Hsieh et al., 2001). The canonical base pair formed by residues 1423C and 1405G is connected to the reverse Hoogsteen interaction between residues 828U (m.2498U) and 835A (m.2508A) of helix H65 (bacterial: *[U1778*·*{A1785]*·(*G2588}:**C2606*); C_v(*U1778*)_ = 1.630, C_v(*A1785*)_ = 1.855, C_v(*G2588*)_ = 1.322, C_v(*C2606*)_ = 1.004)^CbeamM,^^2^ thus contributing to the packing of domains IV and V of 16S mt-rRNA. Position 1424G stacks on top of the 1405G:1423C base pair and shares two hydrogen bonds with position 883A of helix H65 (bacterial: *A1783*·*G2607*; C_v(*A1783*)_ = 1.276, C_v(*G2607*)_ = 0.967)^CbeamM,^^3^ thus mediating the packing of this helix against H93. High-resolution bacterial structures show that the equivalents of the neighbouring residues 1400G and 1402U (bacterial: *G2583* and *U2585*; C_v(*G2583*)_ = 1.969, C_v(*U2585*)_ = 1.942) are within h-bonding distance of A76 of A-site tRNA. Not surprisingly, all mutations at the bacterial position *2585* resulted in dominant lethal phenotypes (Green et al., 1997). In contrast when a G to C mutation was introduced at the bacterial equivalent of position 1424G (m.3094G), position *G2607*, the authors did not report a pronounced growth defect (Xu et al., 2005). Additionally, all mutations at the adjacent universally conserved 1425G (m.3095G) (bacterial *G2608*; C_v(*G2608*)_ = 1.985) were tolerated without significant effects on the growth of *E. coli* cells (Xu et al., 2005). It should be noted that the mutagenesis experiments of Xu et al. were performed in populations carrying hybrid heterogeneous populations of mutant and wild type ribosomes (Xu et al., 2005); a situation reminiscent of mitochondrial heteroplasmy that might have obscured any obvious deleterious effects of the mutations (Xu et al., 2005). This, together with the noted important location of the base change, prompts us not to dismiss 1424G > A (m.3094G > A) as a “likely disruptive” mutation. In the case of the 1423C > G (m.3093C > G) variant, direct biochemical data exists showing impaired mitochondrial activity in skin fibroblasts from the proband (Hsieh et al., 2001). Despite these data, the possibility of a nuclear mutation affecting mitochondrial function has not been clearly ruled out. Taken all the data together, we consider both 1424G > A (m.3094G > A) and the 1423C > G (m.3093C > G) as “likely disruptive” mutations. It should be also noted that resistance to oxazolidinone antibiotics is conferred in bacteria by mutations at position *G2608* (Xu et al., 2005); as such, the possibility that some type of antibiotic hypersusceptibility might be caused by the mutations has to be considered.

## 8 Discussion

The recent publication of medium- and near-atomic resolution cryo-EM structures of the mammalian mitoribosome (Brown et al., 2014) has been used in this report to improve the predictive accuracy of HIA (Smith et al., 2014, Elson et al., 2015). As a result, we have been able to analyse the disruptive potential of 64 very rare human variants mapping to 16S mt-rRNA within their natural structural context. This work is a significant improvement on our previous report on the mutational landscape of the 12S mt-rRNA, which was performed in the absence of homologous high-resolution structures (Smith et al., 2014). However, this new structural knowledge does not decrease the importance of HIA as a tool for unpicking the role of mt-rRNA mutations in disease. This is because despite the major advancement brought about by the achievement of a high-resolution structure of the human mt-LSU, our knowledge of mitochondrial translation lags far behind our understanding of this process in heterologous systems. Thus, we have used in our analysis LSU structures from all three domains of life plus mitochondria (Table 1) to 1) lessen the effects of potential errors in the models during our structural assessment, 2) to have a precise measurement of structural conservation in three dimensions, and 3) to understand the interaction of the human mt-LSU with ligands during translation. Whenever available, published knowledge gathered in heterologous systems has been used to refine, support, and provide a functional rationale to the initial structural analysis. When considering the importance of HIA in this study, the case of the 173U > C (m.1843T > C) mutation should be mentioned (Fig. 2A) (Seibel et al., 2008). The disruptive potential of this mutation can be viewed as “proven” due to the availability of direct biochemical data performed in cybrids (Seibel et al., 2008). Despite the obvious relevance of this mutation, its importance has remained somewhat under appreciated. Besides serving as a perfect control to test the power and accuracy of our methods, showing that the same conclusion regarding the effect of 173U > C (m.1843T > C) would be drawn whether or not the human mt-LSU structure is used for HIA, our analysis has provided a rationale for its mode of action.

Prior to our work, the only mt-rRNA mutations known to affect human health were two relatively mild alterations mapping to SSU mt-rRNA, namely 908A > G (m.1555A > G) and 847C > U (m.1494C > T) (Bindu and Reddy, 2008, Smith et al., 2014). In addition to these mutations, there existed a large number of variants identified in association to human disease but whose potential pathogenicity was supported solely by circumstantial evidence. Our previous paper demonstrated the existence of additional SSU mt-rRNA mutations identified in the context of human disease and/or ageing with the capability to interfere and, in some occasions to completely block the function of the mitoribosomal SSU (Smith et al., 2014). The fact that the two mitoribosomal subunits have distinct, but complementary functions, made it all the more important to answer the question of whether the function of the mitoribosomal LSU rRNA can also be compromised by mutations found associated to human disease and/or ageing. However, proof of the involvement of LSU mt-rRNA component in human disease was yet to be documented, despite the existence of the paper by Seibel et al. (2008). Hence, our goal was to investigate whether LSU mt-rRNA mutations exist with the potential to disrupt mitoribosomal translation. As discussed in our previous paper (Smith et al., 2014), we confined our search for highly disruptive mutations to a limited to a subset of very rare base changes. While this practice would likely leave out some important pathogenic mutations (Smith et al., 2014), it is the most direct way towards the identification of highly disruptive mt-rRNA alterations, those likely to play a role in the disease process. Our results show that, much like in the case of the SSU mt-rRNA, its larger counterpart in the mitoribosome can harbour highly disruptive mutations. Such mutations target the main functional centres of the mitoribosomal LSU such as the PET and the PTC.

One interesting outcome of this study has been the emergence of structural elements associated with the PET of the mammalian mitoribosome, as sites often targeted by potentially pathogenic mutations (Table 1). Two PET mutations identified in cancer samples clearly stand out: the “likely” disruptive 629U > A (m.2299T > A) and the “expectedly” disruptive mutation identified at position 1010 (m.2680) (Table 1, Fig. 2B) (Gochhait et al., 2008, Polyak et al., 1998). Besides these two mutations, eight additional “NEE” mutations map to this functional element (Table 1), particularly to helix H32, which contains five such mutations (yellow box in Fig. 1). While some mutations mapping to the PET have been identified in heterologous organisms (Kloss et al., 2000, Zaman et al., 2007, Vazquez-Laslop et al., 2008, Nakatogawa and Ito, 2002, Lucier et al., 1995), this region of the ribosome has seldom been targeted by mutagenesis analysis. This is especially true for tunnel-associated regions not adjacent to the PTC, such as the structurally conserved helix H32. Given this lack of heterologous evidence, it is not surprising that neither the “likely” nor the “expectedly” categories of Table 1 are populated with H32 mutations. Hence, our results highlight the likely functional importance of this region and prompt for heterologous mutagenesis studies to further assess its role in ribosomal function.

We would like to emphasize that our predictive methods can, for certain mutations, unambiguously establish their disruptive potential, unless a molecular mechanism of translation in mitochondria is proposed that is completely different from that observed in all other domains of life. Such a proposal will be in stark contrast to all the available evidence, particularly with the latest high-resolution mitoribosomal structures, which have confirmed that the universal conservation of the ribosomal core is indeed preserved in the mammalian mitoribosome (Brown et al., 2014). For example, the two P-loop mutations 1145G > A (m.2815G > A) and 1146G > A (m.2816G > A) (Greaves et al., 2010, Taylor et al., 2003, Gaisa et al., 2011) target two adjacent residues known to be essential for the recognition and binding of P-site tRNA (Fig. 2C). Ample biochemical and structural evidence showing the essential nature of the two elements of this binding event, namely the CCA terminus of P-site tRNA and the identity of the equivalents of positions 1145G and 1146G (Samaha et al., 1995, Green et al., 1997, Monro et al., 1968), leads to the inescapable conclusion that the two mitochondrial mutations must seriously disrupt the function of the affected mitoribosomes. A similar case can be made for the 1398G > A (m.3068G > A) base change, known to cause dominant lethal phenotypes in bacteria, a fact that was in agreement with an important role of this residue in the stabilization of the A-site tRNA during peptidyl transfer (Fig. 2D) (Spahn et al., 1996b). Given all this evidence, together with the homoplasmic status of all three mutations, they can almost certainly be considered to be the cause of the COX deficiencies observed in the original samples (Greaves et al., 2010, Taylor et al., 2003, Gaisa et al., 2011).

In addition to these mutations, our studies have revealed a number of “likely” disruptive variants. As initially proposed in our previous paper (Smith et al., 2014), our work has served to prioritize these mutants for additional studies such as heterologous mutagenesis studies that could confirm their suspected disruptive role. One clear example is that of 1169C (m.2839C), which is structurally associated to the P-loop and has been linked to the onset of Dupuytren's disease in a group of patients (Fig. 2C) (Bayat et al., 2005). In Bayat et al.'s study (Bayat et al., 2005), 18 out of 20 patients carried the 1169C > A (m.2839C > A) mutation, suggesting a connection between the mutation and the symptoms observed in the patients. The tight structural connection between this “likely” disruptive site and the functionally crucial P-loop provides a possible functional defect to explain the symptoms observed by Bayat et al. (2005). It should be noted, however, that other studies have ruled out the 1169C > A (m.2839C > A) mutation as the only causative agent of Dupuytren's disease (Anderson et al., 2012). Much like the case of 1169C > A (m.2839C > A), the “likely” disruptive 1420G > A (m.3090G > A) mutation, identified in association with progressive myopathy, is a strong causal candidate of the observed symptoms (Coulbault et al., 2007). Position 1420G is adjacent to the almost invariant 1419A (bacterial: *A2602*; **C**_**v(*A2602*)**_ = **1.939**), which in bacteria is placed between the acceptor ends of P- and A-site tRNAs (Fig. 2E) (Voorhees et al., 2009, Selmer et al., 2006). In both cases, the structural and functional conservation of the involved regions of the PTC, together with the key role in translation of neighbouring residues, makes these sites of mutation obvious targets for heterologous mutagenesis studies that could confirm their disruptive roles.

Finally, we would like to highlight the case of “likely” disruptive 1423C > G (m.3093C > G). This mutation is particularly interesting due to its occurrence with the well-known m.3243A > G mutation, which affects the taurine modification of the wobble position within the anticodon stem loop of mitochondrial tRNA^Leu(UUR)^ and reduces the translation of UUG codons ^(^^Kirino et al., 2004^^)^. The authors hinted at the possibility that the 1423C > G (m.3093C > G) and the m.3243A > G mutation could act in synergy to cause the symptoms observed in the proband (Hsieh et al., 2001). Such synergy could occur if the structural distortion of the PTC, imposed by the 1423C > G (m.3093C > G) mutation (Fig. 2F), effectively amplified the defect introduced by the tRNA alteration. Despite the long distance separating the PTC from the wobble position of tRNA, such an idea would not be too surprising, as it has been amply demonstrated that mutations affecting the structure of the PTC of the LSU can effectively alter the fidelity of decoding in the SSU (Thompson et al., 2001b, O’Connor and Dahlberg, 1995, O'Connor and Dahlberg, 1993).

In summary, our present and previous results (Smith et al., 2014), unequivocally expand the number of mt-rRNA mutations associated with human disease and/or ageing that are capable of severely compromising mitoribosomal function. Taking into account that deleterious mutations have also been identified in nuclear genes encoding protein components of mitoribosomes, mitochondrial translation factors, and elements of the apparatus involved in the maturation of mitoribosomes (Miller et al., 2004, Smits et al., 2011, Serre et al., 1832, Galmiche et al., 2011, Akama et al., 1802, Camara et al., 2011, Dennerlein et al., 2010, Kolanczyk et al., 2011), our results strongly complement an emerging view of the mitoribosome as a key element for human health.

The following are the supplementary data related to this article.

Supplementary material.

**Supplementary Fig. 1:**
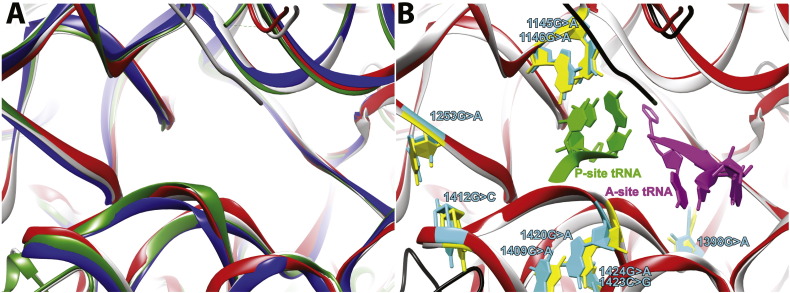
Structure of the peptidyl transferase center (PTC) in LSUs from all 3 domains of life plus mitochondria. A) A ribbon representation of superimposed structures of the PTC from *Homo sapiens* (mitochondria, red), *Thermus thermophilus* (bacteria, grey), *Haloarcula marismortui* (archaea, green), and *Saccharomyces cerevisiae* (eukaryotic cytoplasmic, blue). B) The position of sites of mutation near the PTC is shown for the *H. sapiens* (aquamarine) and *T. thermophilus* (yellow). Sites of mutation described in the text are shown in aquamarine (*H. sapiens* mitoribosomal LSU) and its heterologous counterparts are shown yellow (*T. thermophilus* LSU). The position of A- (light green) and P-site (magenta) tRNAs is shown relative to the sites of mutation. Note that the tRNA structures were resolved in complex with the heterologous *T. thermophilus* LSU.

Description of mutations analyzed in this work.

Description of structures used in this work.

## Conflict of interest

Authors declare no competing financial interests in relation to the work described.

## Figures and Tables

**Table 1:** Classification of mt-LSU rRNA mutations according to their predicted disruptive potential on ribosomal function. The table includes all mitochondrial mutations meeting the criteria described in Methods.

Variation		Canonical base pair partner (*H. sapiens*)	Bacterial equivalent	Canonical base pair partner (bacterial)	Tunnel
NEE					
122G > A	m.1792G > A	115C	G468	C461	Y
161G > A	m.1831G > A	169C	C535	G558	
216G > A	m.1886G > A		U606		
222A > G	m.1892A > G				
235C > A	m.C1905C > A		C673		Y
243G > A	m.1913G > A	336C	G681	C796	Y
270A > G	m.1940A > G	264U	C730	G702	
282U > C	m.T1952T > C	295A	G742	C755	
297U > C	m.1967T > C	280U	U757	U740	
320G > A	m.1990G > A		G780		Y
334G > A	m.2004G > A	245C	794	683	Y
337U > C	m.2007T > C	242A	C797	G680	Y
345G > A	m.2015G > A		G805		Y
404delA	m.2074delA				
409C > U	m.2079C > T				
413U > G	m.2083T > G				
449 3T > 4T	m.2119 3T > 4T	432A	U967	G950	
451G > A	m.2121G > A	430C	G969	C948	
478–479 dupAG	m.2148–2149 dupAG	582C & 583U	G997 & C998	G1157 & C1158	
480 delU	m.2150 delT	?	U999	A1155	
481 delA	m.2151 delA		A1000		
605U > C	m.2275T > C	620A	U1199	A1246	
880A > U	m.2550A > T		G1839		
948U > C	m.2618T > C		U1955		
973G > C	m.2643G > C		A1980	C1771	
974A > G	m.2644A > G		A1981		
994U > C	m.2664T > C	793A	C2001	G1659	
1028G > A	m.2698G > A		G2035		Y
1135A > U	m.2805A > T	1072U	A2241	U2079	
1283U > C	m.2953T > C	1301A	C2466	G2484	
1328 InsU	m.2998 InsT		U2511		
1344G > A	m.3014G > A	1353C	C2527	G2536	
1362G > A	m.3032G > A	1337C	G2545	C2520	
1384G > A	m.3054G > A	1334C	G2567	C2517	
1490A > U	m.3160A > T				
1535C > U	m.3205C > T				
Unlikely					
34U > C	m.1704T > C				
253C > U	m.1923C > T	311G	C691	G771	
568A > G	m.2238A > G				
677C > U	m.2347C > T	696G			
1011G > A	m.2681G > A	157C	G2018	C531	
1433C > U	m.3103C > U	1046G	C2616	G2053	
Undetermined					
399U > G	m.2069T > G				
551C > U	m.2221C > T				
1114A > C	m.2784A > C				
1120 InsT	m.2790 InsT				
1214C > U	m.2884C > T				
Likely					
629U > A	m.2299T > A		U1255		
875U > C	m.2545T > C		U1834		
889U > G	m.2559T > G				
923G > A	m.2593G > A		G1930		
1169C > A	m.2839C > A	1149G	C2275	G2255	
1253G > A	m.2923G > A	1066C	G2436	C2073	
1328U > C	m.2998T > C		U2511		
1409G > A	m.3079G > A	1418C	G2592	C2601	
1412G > C	m.3082G > C		G2595		
1420G > A	m.3090G > A	1408C	G2603	C2591	
1423C > G	m.3093C > G	1405G	C2606	G2588	
1424G > A	m.3094G > A		G2607		
Expectedly					
1010U > C	m.2680T > C	636A	U2017	A1262	Y
1145G > A	m.2815G > A		G2251		
1146G > A	m.2816G > A		G2252		
1398G > A	m.3068G > A		G2581		
Proven					
173U > C	m.1843T > C	1026A	U562	A2033	

## Footnotes

2 Some distances are slightly longer on the *Saccharomyces cerevisiae* structure. The archaeal equivalent of position 835A (bacteria: *A1785*) is C1841. Despite this substitution, the four-base interaction is maintained in *Haloarcula marismortui*.

3 The *S. cerevisiae* equivalent of 833A (bacterial: *A1783*) is *U2141*. As a result, one of the two hydrogen bonds is lost.

## References

[bb0005] Aagaard C., Douthwaite S. (1994). Requirement for a conserved, tertiary interaction in the core of 23S ribosomal RNA. Proc. Natl. Acad. Sci..

[bb0010] Akama K., Christian B.E., Jones C.N., Ueda T., Takeuchi N., Spremulli L.L. (1802). Analysis of the functional consequences of lethal mutations in mitochondrial translational elongation factors. Biochim. Biophys. Acta.

[bb0015] Ali I.K., Lancaster L., Feinberg J., Joseph S., Noller H.F. (2006). Deletion of a conserved, central ribosomal intersubunit RNA bridge. Mol. Cell.

[bb0020] Anderson E.R., Burmester J.K., Caldwell M.D. (2012). Evaluation of a mitochondrial DNA mutation in maternally inherited and sporadic cases of Dupuytren disease. Clin. Med. Res..

[bb0025] Andrews R.M., Kubacka I., Chinnery P.F., Lightowlers R.N., Turnbull D.M., Howell N. (1999). Reanalysis and revision of the Cambridge reference sequence for human mitochondrial DNA. Nat. Genet..

[bb0030] Bayat A., Walter J., Lambe H. (2005). Identification of a novel mitochondrial mutation in Dupuytren's disease using multiplex DHPLC. Plast. Reconstr. Surg..

[bb0035] Belanger F., Gagnon M.G., Steinberg S.V., Cunningham P.R., Brakier-Gingras L. (2004). Study of the functional interaction of the 900 tetraloop of 16S ribosomal RNA with helix 24 within the bacterial ribosome. J. Mol. Biol..

[bb0040] Bindu L.H., Reddy P.P. (2008). Genetics of aminoglycoside-induced and prelingual non-syndromic mitochondrial hearing impairment: a review. Int. J. Audiol..

[bb0045] Brown A., Amunts A., Bai X.C. (2014). Structure of the large ribosomal subunit from human mitochondria. Science.

[bb0050] Camara Y., Asin-Cayuela J., Park C.B. (2011). MTERF4 regulates translation by targeting the methyltransferase NSUN4 to the mammalian mitochondrial ribosome. Cell Metab..

[bb0055] Cannone J.J., Subramanian S., Schnare M.N. (2002). The comparative RNA web (CRW) site: an online database of comparative sequence and structure information for ribosomal, intron, and other RNAs. BMC Bioinforma..

[bb0060] Coulbault L., Deslandes B., Herlicoviez D. (2007). A novel mutation 3090G > A of the mitochondrial 16S ribosomal RNA associated with myopathy. Biochem. Biophys. Res. Commun..

[bb0065] Dennerlein S., Rozanska A., Wydro M., Chrzanowska-Lightowlers Z.M., Lightowlers R.N. (2010). Human ERAL1 is a mitochondrial RNA chaperone involved in the assembly of the 28S small mitochondrial ribosomal subunit. Biochem. J..

[bb0070] Elson J.L., Samuels D.C., Turnbull D.M., Chinnery P.F. (2001). Random intracellular drift explains the clonal expansion of mitochondrial DNA mutations with age. Am. J. Hum. Genet..

[bb0075] Elson J.L., Smith P.M., Vila-Sanjurjo A. (2015). Heterologous inferential analysis (HIA) as a method to understand the role of mitochondrial rRNA mutations in pathogenesis. Methods Mol. Biol..

[bb0080] Elson J.L., Swalwell H., Blakely E.L., McFarland R., Taylor R.W., Turnbull D.M. (2009). Pathogenic mitochondrial tRNA mutations — which mutations are inherited and why?. Hum. Mutat..

[bb0085] Feinberg J.S., Joseph S. (2006). A conserved base-pair between tRNA and 23S rRNA in the peptidyl transferase center is important for peptide release. J. Mol. Biol..

[bb0090] Fendt L., Niederstatter H., Huber G. (2011). Accumulation of mutations over the entire mitochondrial genome of breast cancer cells obtained by tissue microdissection. Breast Cancer Res. Treat..

[bb0095] Gaisa N.T., Graham T.A., McDonald S.A. (2011). The human urothelium consists of multiple clonal units, each maintained by a stem cell. J. Pathol..

[bb0100] Galmiche L., Serre V., Beinat M. (2011). Exome sequencing identifies MRPL3 mutation in mitochondrial cardiomyopathy. Hum. Mutat..

[bb0105] Ghosh S., Joseph S. (2005). Nonbridging phosphate oxygens in 16S rRNA important for 30S subunit assembly and association with the 50S ribosomal subunit. RNA.

[bb0110] Gochhait S., Bhatt A., Sharma S., Singh Y.P., Gupta P., Bamezai R.N. (2008). Concomitant presence of mutations in mitochondrial genome and p53 in cancer development — a study in north Indian sporadic breast and esophageal cancer patients. Int. J. Cancer.

[bb0115] Gorman G.S., Schaefer A.M., Ng Y. (2015). Prevalence of nuclear and mtDNA mutations related to adult mitochondrial disease. Ann. Neurol..

[bb0120] Greaves L.C., Barron M.J., Plusa S. (2010). Defects in multiple complexes of the respiratory chain are present in ageing human colonic crypts. Exp. Gerontol..

[bb0125] Greber B.J., Boehringer D., Leitner A. (2014). Architecture of the large subunit of the mammalian mitochondrial ribosome. Nature.

[bb0130] Green R., Samaha R.R., Noller H.F. (1997). Mutations at nucleotides G2251 and U2585 of 23S rRNA perturb the peptidyl transferase center of the ribosome. J. Mol. Biol..

[bb0135] Hsieh R.H., Li J.Y., Pang C.Y., Wei Y.H. (2001). A novel mutation in the mitochondrial 16S rRNA gene in a patient with MELAS syndrome, diabetes mellitus, hyperthyroidism and cardiomyopathy. J. Biomed. Sci..

[bb0140] Jeronimo C., Nomoto S., Caballero O.L. (2001). Mitochondrial mutations in early stage prostate cancer and bodily fluids. Oncogene.

[bb0145] Katoh K., Misawa K., Kuma K., Miyata T. (2002). MAFFT: a novel method for rapid multiple sequence alignment based on fast Fourier transform. Nucleic Acids Res..

[bb0150] Kaushal P.S., Sharma M.R., Booth T.M. (2014). Cryo-EM structure of the small subunit of the mammalian mitochondrial ribosome. Proc. Natl. Acad. Sci. U. S. A..

[bb0155] Kim H.M., Yeom J.H., Ha H.J., Kim J.M., Lee K. (2007). Functional analysis of the residues C770 and G771 of *E. coli* 16S rRNA implicated in forming the intersubunit bridge B2c of the ribosome. J. Microbiol. Biotechnol..

[bb0160] Kirino Y., Yasukawa T., Ohta S. (2004). Codon-specific translational defect caused by a wobble modification deficiency in mutant tRNA from a human mitochondrial disease. Proc. Natl. Acad. Sci. U. S. A..

[bb0165] Kloss-Brandstatter A., Schafer G., Erhart G. (2010). Somatic mutations throughout the entire mitochondrial genome are associated with elevated PSA levels in prostate cancer patients. Am. J. Hum. Genet..

[bb0170] Koc E.C., Haque M.E., Spremulli L.L. (2010). Current views of the structure of the mammalian mitochondrial ribosome. Isr. J. Chem..

[bb0175] Kolanczyk M., Pech M., Zemojtel T. (2011). NOA1 is an essential GTPase required for mitochondrial protein synthesis. Mol. Biol. Cell.

[bb0180] Leviev I.G., Rodriguez-Fonseca C., Phan H. (1994). A conserved secondary structural motif in 23S rRNA defines the site of interaction of amicetin, a universal inhibitor of peptide bond formation. EMBO J..

[bb0185] Lieberman K.R., Dahlberg A.E. (1994). The importance of conserved nucleotides of 23S ribosomal RNA and transfer RNA in ribosome catalyzed peptide bond formation. J. Biol. Chem..

[bb0190] Liiv A., O'Connor M. (2006). Mutations in the intersubunit bridge regions of 23S rRNA. J. Biol. Chem..

[bb0195] Liu J., Wang L.D., Sun Y.B. (2012). Deciphering the signature of selective constraints on cancerous mitochondrial genome. Mol. Biol. Evol..

[bb0200] Lloyd R.E., McGeehan J.E. (2013). Structural analysis of mitochondrial mutations reveals a role for bigenomic protein interactions in human disease. PLoS One.

[bb0205] Long K.S., Munck C., Andersen T.M. (2010). Mutations in 23S rRNA at the peptidyl transferase center and their relationship to linezolid binding and cross-resistance. Antimicrob. Agents Chemother..

[bb0210] Lorenc A., Bryk J., Golik P. (2003). Homoplasmic MELAS A3243G mtDNA mutation in a colon cancer sample. Mitochondrion.

[bb0215] Lucier T.S., Heitzman K., Liu S.K., Hu P.C. (1995). Transition mutations in the 23S rRNA of erythromycin-resistant isolates of *Mycoplasma pneumoniae*. Antimicrob. Agents Chemother..

[bb0220] McDonald S.A., Greaves L.C., Gutierrez-Gonzalez L. (2008). Mechanisms of field cancerization in the human stomach: the expansion and spread of mutated gastric stem cells. Gastroenterology.

[bb0225] Miller C., Saada A., Shaul N. (2004). Defective mitochondrial translation caused by a ribosomal protein (MRPS16) mutation. Ann. Neurol..

[bb0230] Mitchell A.L., Elson J.L., Howell N., Taylor R.W., Turnbull D.M. (2006). Sequence variation in mitochondrial complex I genes: mutation or polymorphism?. J. Med. Genet..

[bb0235] Mithani S.K., Taube J.M., Zhou S. (2007). Mitochondrial mutations are a late event in the progression of head and neck squamous cell cancer. Clin. Cancer Res..

[bb0240] Monro R.E., Cerna J., Marcker K.A. (1968). Ribosome-catalyzed peptidyl transfer: substrate specificity at the P-site. Proc. Natl. Acad. Sci. U. S. A..

[bb0245] Nakatogawa H., Ito K. (2002). The ribosomal exit tunnel functions as a discriminating gate. Cell.

[bb0250] Nissen P., Hansen J., Ban N., Moore P.B., Steitz T.A. (2000). The structural basis of ribosome activity in peptide bond synthesis. Science.

[bb0255] O'Connor M., Dahlberg A.E. (1995). The involvement of two distinct regions of 23S ribosomal RNA in tRNA selection. J. Mol. Biol..

[bb0260] O'Connor M. (2007). Selection for intragenic suppressors of lethal 23S rRNA mutations in *Escherichia coli* identifies residues important for ribosome assembly and function. Mol. Gen. Genomics..

[bb0265] O'Connor M., Dahlberg A.E. (1993). Mutations at U2555, a tRNA-protected base in 23S rRNA, affect translational fidelity. Proc. Natl. Acad. Sci. U. S. A..

[bb0270] Osterman I.A., Sergiev P.V., Tsvetkov P.O., Makarov A.A., Bogdanov A.A., Dontsova O.A. (2011). Methylated 23S rRNA nucleotide m2G1835 of *Escherichia coli* ribosome facilitates subunit association. Biochimie.

[bb0275] Pan B., Mitra S.N., Sundaralingam M. (1998). Structure of a 16-mer RNA duplex r(GCAGACUUAAAUCUGC)2 with wobble C·A + mismatches. J. Mol. Biol..

[bb0280] Pechmann S., Willmund F., Frydman J. (2013). The ribosome as a hub for protein quality control. Mol. Cell.

[bb0285] Pereira L., Freitas F., Fernandes V. (2009). The diversity present in 5140 human mitochondrial genomes. Am. J. Hum. Genet..

[bb0290] Petrov A.S., Bernier C.R., Hsiao C. (2014). Evolution of the ribosome at atomic resolution. Proc. Natl. Acad. Sci. U. S. A..

[bb0295] Pettersen E.F., Goddard T.D., Huang C.C. (2004). UCSF Chimera — a visualization system for exploratory research and analysis. J. Comput. Chem..

[bb0300] Polacek N., Gomez M.J., Ito K., Xiong L., Nakamura Y., Mankin A. (2003). The critical role of the universally conserved A2602 of 23S ribosomal RNA in the release of the nascent peptide during translation termination. Mol. Cell.

[bb0305] Polyak K., Li Y., Zhu H. (1998). Somatic mutations of the mitochondrial genome in human colorectal tumours. Nat. Genet..

[bb0310] Porse B.T., Thi-Ngoc H.P., Garrett R.A. (1996). The donor substrate site within the peptidyl transferase loop of 23S rRNA and its putative interactions with the CCA-end of N-blocked aminoacyl-tRNA^Phe^. J. Mol. Biol..

[bb0315] Rackham O., Wang K., Chin J.W. (2006). Functional epitopes at the ribosome subunit interface. Nat. Chem. Biol..

[bb0320] Ruiz-Pesini E., Lott M.T., Procaccio V. (2007). An enhanced MITOMAP with a global mtDNA mutational phylogeny. Nucleic Acids Res..

[bb0325] Saenger W. (1984). Principles of Nucleic Acid Structure.

[bb0330] Samaha R.R., Green R., Noller H.F. (1995). A base pair between tRNA and 23S rRNA in the peptidyl transferase centre of the ribosome. Nature.

[bb0335] Sayers E. (2010). E-Utilities Quick Start. Anonymous Entrez Programming Utilities Help [Internet].

[bb0340] Scheffler I.E. (1999). Mitochondria.

[bb0345] Seibel P., Di Nunno C., Kukat C. (2008). Cosegregation of novel mitochondrial 16S rRNA gene mutations with the age-associated T414G variant in human cybrids. Nucleic Acids Res..

[bb0350] Selmer M., Dunham C.M., Murphy F.V. (2006). Structure of the 70S ribosome complexed with mRNA and tRNA. Science.

[bb0355] Serre V., Rozanska A., Beinat M. (1832). Mutations in mitochondrial ribosomal protein MRPL12 leads to growth retardation, neurological deterioration and mitochondrial translation deficiency. Biochim. Biophys. Acta.

[bb0360] Smith P.M., Elson J.L., Greaves L.C. (2014). The role of the mitochondrial ribosome in human disease: searching for mutations in 12S mitochondrial rRNA with high disruptive potential. Hum. Mol. Genet..

[bb0365] Smits P., Saada A., Wortmann S.B. (2011). Mutation in mitochondrial ribosomal protein MRPS22 leads to Cornelia de Lange-like phenotype, brain abnormalities and hypertrophic cardiomyopathy. Eur. J. Hum. Genet..

[bb0370] Spahn C.M., Remme J., Schäfer M.A., Nierhaus K.H. (1996). Mutational analysis of two highly conserved UGG sequences of 23S rRNA from *Escherichia coli*. J. Biol. Chem..

[bb0375] Spahn C.M., Schäfer M.A., Krayevsky A.A., Nierhaus K.H. (1996). Conserved nucleotides of 23S rRNA located at the ribosomal peptidyltransferase center. J. Biol. Chem..

[bb0380] Stewart J.B., Freyer C., Elson J.L. (2008). Strong purifying selection in transmission of mammalian mitochondrial DNA. PLoS Biol..

[bb0385] Taylor R.W., Barron M.J., Borthwick G.M. (2003). Mitochondrial DNA mutations in human colonic crypt stem cells. J. Clin. Invest..

[bb0390] Thompson J., Kim D.F., O'Connor M. (2001). Analysis of mutations at residues A2451 and G2447 of 23S rRNA in the peptidyltransferase active site of the 50S ribosomal subunit. Proc. Natl. Acad. Sci. U. S. A..

[bb0395] Thompson J., Tapprich W.E., Munger C., Dahlberg A.E. (2001). *Staphylococcus aureus* domain V functions in *Escherichia coli* ribosomes provided a conserved interaction with domain IV is restored. RNA.

[bb0400] Tuppen H.A., Blakely E.L., Turnbull D.M., Taylor R.W. (1797). Mitochondrial DNA mutations and human disease. Biochim. Biophys. Acta.

[bb0405] Vazquez-Laslop N., Thum C., Mankin A.S. (2008). Molecular mechanism of drug-dependent ribosome stalling. Mol. Cell.

[bb0410] Voorhees R.M., Weixlbaumer A., Loakes D., Kelley A.C., Ramakrishnan V. (2009). Insights into substrate stabilization from snapshots of the peptidyl transferase center of the intact 70S ribosome. Nat. Struct. Mol. Biol..

[bb0415] Wallace D.C. (2010). Mitochondrial DNA mutations in disease and aging. Environ. Mol. Mutagen..

[bb0420] Xiong L., Kloss P., Douthwaite S. (2000). Oxazolidinone resistance mutations in 23S rRNA of *Escherichia coli* reveal the central region of domain V as the primary site of drug action. J. Bacteriol..

[bb0425] Xu J., Golshani A., Aoki H. (2005). Protected nucleotide G2608 in 23S rRNA confers resistance to oxazolidinones in *E. coli*. Biochem. Biophys. Res. Commun..

[bb0430] Yarham J.W., Al-Dosary M., Blakely E.L. (2011). A comparative analysis approach to determining the pathogenicity of mitochondrial tRNA mutations. Hum. Mutat..

[bb0435] Youngman E.M., Brunelle J.L., Kochaniak A.B., Green R. (2004). The active site of the ribosome is composed of two layers of conserved nucleotides with distinct roles in peptide bond formation and peptide release. Cell.

[bb0440] Yusupov M.M., Yusupova G.Z., Baucom A. (2001). Crystal structure of the ribosome at 5.5 A resolution. Science.

[bb0445] Zaman S., Fitzpatrick M., Lindahl L., Zengel J. (2007). Novel mutations in ribosomal proteins L4 and L22 that confer erythromycin resistance in *Escherichia coli*. Mol. Microbiol..

[bb0450] Zhou S., Kachhap S., Sun W. (2007). Frequency and phenotypic implications of mitochondrial DNA mutations in human squamous cell cancers of the head and neck. Proc. Natl. Acad. Sci. U. S. A..

