# Supplementary material for: The presence of highly disruptive 16S rRNA mutations in clinical samples indicates a wider role for mutations of the mitochondrial ribosome in human disease
Source: Mitochondrion. 2015 Nov;25:17–27. doi: 10.1016/j.mito.2015.08.004 (PMC4665369; doi:10.1016/j.mito.2015.08.004)
Supplement: Supplementary file 1 — Supplementary material. [file mmc1.doc]

**SUPPLEMENTAL MATERIAL**

**The presence of highly disruptive 16S rRNA mutations in clinical samples indicates a wider role for mutations of the mitochondrial ribosome in human disease**

**Joanna L. Elson^@,1,2^, Paul M. Smith^@,3,4^, Laura C. Greaves^4^, Robert N. Lightowlers^5^, Zofia M. A. Chrzanowska-Lightowlers^4^, Robert W. Taylor^4^, Antón Vila-Sanjurjo^6,*^**

@these authors contributed equally to this work

**^1^**Institute of Genetic Medicine, Newcastle University, Newcastle upon Tyne, United Kingdom, NE1 3BZ, United Kingdom.

^2^Centre for Human Metabonomics, North-West University, Potchefstroom, South Africa.

^3^Institute of Medical Sciences, Ninewells Hospital and Medical School, Dundee University, Dundee, Scotland. UK, DD1 9SY.

^4^Wellcome Trust Centre for Mitochondrial Research, Institute of Neuroscience, Newcastle University, The Medical School, Newcastle upon Tyne, NE2 4HH, United Kingdom.

^5^Newcastle University Institute for Cell and Molecular Biosciences, Newcastle University, The Medical School, Newcastle upon Tyne, NE2 4HH, United Kingdom.

**^6^**Grupo GIBE, Bioloxía Celular e Molecular, Facultade de Ciencias, Universidade da Coruña (UDC). 15071 A Coruña, Spain.

^*^To whom correspondence should be addressed at Grupo GIBE, Bioloxía Celular e Molecular, Facultade de Ciencias, Universidade da Coruña (UDC). 15071 A Coruña, Spain. Tel: +34 981 167000 ext:2659; Fax: +34 981 167068; Email: [antonvila.s@gmail.com](mailto:antonvila.s@gmail.com).

**ALIGNMENT BETWEEN RIBOSOMAL PROTEINS L4 (*Thermus thermophilus*) AND MRPL4 (*Homo sapienS*)**

***Thermus thermophilus L4***

>gi|62287203|sp|Q5SHN9.1|RL4_THET8 RecName: Full=50S ribosomal protein L4; AltName: Full=L1e

MKEVAVYQIPVLSPSGRRELAADLPAEINPHLLWEVVRWQLAKRRRGTASTKTRGEVAYSGRKIWPQKHT

GRARHGDIGAPIFVGGGVVFGPKPRDYSYTLPKKVRKKGLAMAVADRAREGKLLLVEAFAGVNGKTKEFL

AWAKEAGLDGSESVLLVTGNELVRRAARNLPWVVTLAPEGLNVYDIVRTERLVMDLDAWEVFQNRIGGEA

***Homo sapiens MRPL4***

>gi|22547138|ref|NP_666499.1| 39S ribosomal protein L4, mitochondrial isoform a [Homo sapiens]

MLQFVRAGARAWLRPTGSQGLSSLAEEAARATENPEQVASEGLPEPVLRKVELPVPTHRRPVQAWVESLR

GFEQERVGLADLHPDVFATAPRLDILHQVAMWQKNFKRISYAKTKTRAEVRGGGRKPWPQKGTGRARHGS

IRSPLWRGGGVAHGPRGPTSYYYMLPMKVRALGLKVALTVKLAQDDLHIMDSLELPTGDPQYLTELAHYR

RWGDSVLLVDLTHEEMPQSIVEATSRLKTFNLIPAVGLNVHSMLKHQTLVLTLPTVAFLEDKLLWQDSRY

RPLYPFSLPYSDFPRPLPHATQGPAATPYHC

L4 210 bp 210 aa vs.

MRPL4 311 bp 311 aa

using matrix file: BLOSUM50, gap open/ext: -14/-4

21.5% identity in 311 aa overlap; Global score: 269

L4 ----------------------------------------------------------MK

.

MRPL4 MLQFVRAGARAWLRPTGSQGLSSLAEEAARATENPEQVASEGLPEPVLRKVELPVPTHRR

10 20 30 40 50 60

10 20 30 40 50

L4 EVAVYQIPVLSPSGRRELAADLPAEI---NPHL--LWEVVRWQLAKRRRGTASTKTRGEV

: .. . . .: ::: .. :.: : .:. :: .: . :.::::.::

MRPL4 PVQAWVESLRGFEQERVGLADLHPDVFATAPRLDILHQVAMWQKNFKRISYAKTKTRAEV

70 80 90 100 110 120

60 70 80 90 100 110

L4 AYSGRKIWPQKHTGRARHGDIGAPIFVGGGVVFGPK-PRDYSYTLPKKVRKKGLAMAVAD

.::: :::: :::::::.: .:.. ::::. ::. : .: : :: ::: :: .:..

MRPL4 RGGGRKPWPQKGTGRARHGSIRSPLWRGGGVAHGPRGPTSYYYMLPMKVRALGLKVALTV

130 140 150 160 170 180

120 130 140 150 160 170

L4 RAREGKLLLVEAFAGVNGKTKEFLAWAKEAGLDGSESVLLVTGNEL---VRRAARNLPWV

. . : ..... .: . . :. : .. .: .:. . .:. :

MRPL4 KLAQDDLHIMDSLELPTGDPQYLTELAHYRRWGDSVLLVDLTHEEMPQSIVEATSRLKTF

190 200 210 220 230 240

180 190 200 210

L4 VTLAPEGLNVYDIVRTERLVMDLDAWEVFQNRIGGEA-----------------------

. ::::..... . ::. : . ..... .

MRPL4 NLIPAVGLNVHSMLKHQTLVLTLPTVAFLEDKLLWQDSRYRPLYPFSLPYSDFPRPLPHA

250 260 270 280 290 300

L4 -----------

MRPL4 TQGPAATPYHC

310

Alignment was performed with Lalign ^1^.

**Supplemental Figure 1:** Structure of the peptidyl transferase center (PTC) in LSUs from all 3 domains of life plus mitochondria. **A)** A ribbon representation of superimposed structures of the PTC from *Homo sapiens* (mitochondria, red), *Thermus thermophilus* (bacteria, grey), *Haloarcula marismortui* (archaea, green), and *Saccharomyces cerevisiae* (eukaryotic cytoplasmic, blue). **B)** The position of sites of mutation near the PTC are shown for the *H. sapiens* (aquamarine) and *T. thermophilus* (yellow). Sites of mutation described in the text are shown in aquamarine (*H. sapiens* mitoribosomal LSU) and its heterologous counterparts are shown yellow (*T. thermophilus* LSU). The position of A- (light green) and P-site (magenta) tRNAs is shown relative to the sites of mutation. Note that the tRNA structures were resolved in complex with the heterologous *T. thermophilus* LSU.

**REFERENCES**

1 Huang X, Miller W: A time-efficient, linear-space local similarity algorithm. *Advances in Applied Mathematics* 1991; **12:** 337-357.

**References Suppl. Table I**

1 Gaisa NT, Graham TA, McDonald SA *et al:* The human urothelium consists of multiple clonal units, each maintained by a stem cell. *J Pathol* 2011; **225:** 163-171.

2 Gomez-Zaera M, Abril J, Gonzalez L *et al:* Identification of somatic and germline mitochondrial DNA sequence variants in prostate cancer patients. *Mutat Res* 2006; **595:** 42-51.

3 Taylor RW, Barron MJ, Borthwick GM *et al:* Mitochondrial DNA mutations in human colonic crypt stem cells. *J Clin Invest* 2003; **112:** 1351-1360.

4 Seibel P, Di Nunno C, Kukat C *et al:* Cosegregation of novel mitochondrial 16S rRNA gene mutations with the age-associated T414G variant in human cybrids. *Nucleic Acids Res* 2008; **36:** 5872-5881.

5 Freitag M, Holger P, Meitinger T: MITOMAP mtDNA Sequence Data: Unpublished Variant 20110302018. 2011; .

6 Guo LJ, Oshida Y, Fuku N *et al:* Mitochondrial genome polymorphisms associated with type-2 diabetes or obesity. *Mitochondrion* 2005; **5:** 15-33.

7 Greaves LC, Barron MJ, Plusa S *et al:* Defects in multiple complexes of the respiratory chain are present in ageing human colonic crypts. *Exp Gerontol* 2010; **45:** 573-579.

8 Tseng LM, Yin PH, Yang CW *et al:* Somatic mutations of the mitochondrial genome in human breast cancers. *Genes Chromosomes Cancer* 2011; **50:** 800-811.

9 Nagy A, Wilhelm M, Sukosd F, Ljungberg B, Kovacs G: Somatic mitochondrial DNA mutations in human chromophobe renal cell carcinomas. *Genes Chromosomes Cancer* 2002; **35:** 256-260.

10 Liu VW, Shi HH, Cheung AN *et al:* High incidence of somatic mitochondrial DNA mutations in human ovarian carcinomas. *Cancer Res* 2001; **61:** 5998-6001.

11 Polyak K, Li Y, Zhu H *et al:* Somatic mutations of the mitochondrial genome in human colorectal tumours. *Nat Genet* 1998; **20:** 291-293.

12 Zhou S, Kachhap S, Sun W *et al:* Frequency and phenotypic implications of mitochondrial DNA mutations in human squamous cell cancers of the head and neck. *Proc Natl Acad Sci U S A* 2007; **104:** 7540-7545.

13 Mithani SK, Taube JM, Zhou S *et al:* Mitochondrial mutations are a late event in the progression of head and neck squamous cell cancer. *Clin Cancer Res* 2007; **13:** 4331-4335.

14 Kloss-Brandstatter A, Schafer G, Erhart G *et al:* Somatic mutations throughout the entire mitochondrial genome are associated with elevated PSA levels in prostate cancer patients. *Am J Hum Genet* 2010; **87:** 802-812.

15 Jones JB, Song JJ, Hempen PM, Parmigiani G, Hruban RH, Kern SE: Detection of mitochondrial DNA mutations in pancreatic cancer offers a "mass"-ive advantage over detection of nuclear DNA mutations. *Cancer Res* 2001; **61:** 1299-1304.

16 Wani AA, Ahanger SH, Bapat SA *et al:* Analysis of mitochondrial DNA sequences in childhood encephalomyopathies reveals new disease-associated variants. *PLoS One* 2007; **2:** e942.

17 Liu J, Wang LD, Sun YB *et al:* Deciphering the signature of selective constraints on cancerous mitochondrial genome. *Mol Biol Evol* 2012; **29:** 1255-1261.

18 Bi R, Li WL, Chen MQ, Zhu Z, Yao YG: Rapid identification of mtDNA somatic mutations in gastric cancer tissues based on the mtDNA phylogeny. *Mutat Res* 2011; **709-710:** 15-20.

19 Zhao L, Young WY, Li R, Wang Q, Qian Y, Guan MX: Clinical evaluation and sequence analysis of the complete mitochondrial genome of three Chinese patients with hearing impairment associated with the 12S rRNA T1095C mutation. *Biochem Biophys Res Commun* 2004; **325:** 1503-1508.

20 Wang CY, Wang HW, Yao YG, Kong QP, Zhang YP: Somatic mutations of mitochondrial genome in early stage breast cancer. *Int J Cancer* 2007; **121:** 1253-1256.

21 Greaves LC, Elson JL, Nooteboom M *et al:* Comparison of mitochondrial mutation spectra in ageing human colonic epithelium and disease: absence of evidence for purifying selection in somatic mitochondrial DNA point mutations. *PLoS Genet* 2012; **8:** e1003082.

22 McDonald SA, Greaves LC, Gutierrez-Gonzalez L *et al:* Mechanisms of field cancerization in the human stomach: the expansion and spread of mutated gastric stem cells. *Gastroenterology* 2008; **134:** 500-510.

23 Subhankar B, Dhananjaya S: MITOMAP mtDNA Sequence Data: Unpublished Variant 20041220003. 2003; .

24 Fliss MS, Usadel H, Caballero OL *et al:* Facile detection of mitochondrial DNA mutations in tumors and bodily fluids. *Science* 2000; **287:** 2017-2019.

25 Gochhait S, Bhatt A, Sharma S, Singh YP, Gupta P, Bamezai RN: Concomitant presence of mutations in mitochondrial genome and p53 in cancer development - a study in north Indian sporadic breast and esophageal cancer patients. *Int J Cancer* 2008; **123:** 2580-2586.

26 Witte J, Lehmann S, Wulfert M, Yang Q, Roher HD: Mitochondrial DNA mutations in differentiated thyroid cancer with respect to the age factor. *World J Surg* 2007; **31:** 51-59.

27 Kassauei K, Habbe N, Mullendore ME, Karikari CA, Maitra A, Feldmann G: Mitochondrial DNA mutations in pancreatic cancer. *Int J Gastrointest Cancer* 2006; **37:** 57-64.

28 Tanwar M, Dada T, Sihota R, Dada R: Mitochondrial DNA analysis in primary congenital glaucoma. *Mol Vis* 2010; **16:** 518-533.

29 Bayat A, Walter J, Lambe H *et al:* Identification of a novel mitochondrial mutation in Dupuytren's disease using multiplex DHPLC. *Plast Reconstr Surg* 2005; **115:** 134-141.

30 Jeronimo C, Nomoto S, Caballero OL *et al:* Mitochondrial mutations in early stage prostate cancer and bodily fluids. *Oncogene* 2001; **20:** 5195-5198.

31 Fendt L, Niederstatter H, Huber G *et al:* Accumulation of mutations over the entire mitochondrial genome of breast cancer cells obtained by tissue microdissection. *Breast Cancer Res Treat* 2011; **128:** 327-336.

32 Lorenc A, Bryk J, Golik P *et al:* Homoplasmic MELAS A3243G mtDNA mutation in a colon cancer sample. *Mitochondrion* 2003; **3:** 119-124.

33 Coulbault L, Deslandes B, Herlicoviez D *et al:* A novel mutation 3090 G>A of the mitochondrial 16S ribosomal RNA associated with myopathy. *Biochem Biophys Res Commun* 2007; **362:** 601-605.

**References Suppl. Table II**

1 Brown A, Amunts A, Bai XC *et al:* Structure of the large ribosomal subunit from human mitochondria. *Science* 2014; **346:** 718-722.

2 Greber BJ, Boehringer D, Leitner A *et al:* Architecture of the large subunit of the mammalian mitochondrial ribosome. *Nature* 2014; **505:** 515-519.

3 Kaushal PS, Sharma MR, Booth TM *et al:* Cryo-EM structure of the small subunit of the mammalian mitochondrial ribosome. *Proc Natl Acad Sci U S A* 2014; **111:** 7284-7289.

4 Amunts A, Brown A, Bai XC *et al:* Structure of the yeast mitochondrial large ribosomal subunit. *Science* 2014; **343:** 1485-1489.

5 Gabdulkhakov A, Nikonov S, Garber M: Revisiting the Haloarcula marismortui 50S ribosomal subunit model. *Acta Crystallogr D Biol Crystallogr* 2013; **69:** 997-1004.

6 Pulk A, Cate JH: Control of ribosomal subunit rotation by elongation factor G. *Science* 2013; **340:** 1235970.

7 Selmer M, Dunham CM, Murphy FV,4th *et al:* Structure of the 70S ribosome complexed with mRNA and tRNA. *Science* 2006; **313:** 1935-1942.

8 Gao YG, Selmer M, Dunham CM, Weixlbaumer A, Kelley AC, Ramakrishnan V: The structure of the ribosome with elongation factor G trapped in the posttranslocational state. *Science* 2009; **326:** 694-699.

9 Voorhees RM, Weixlbaumer A, Loakes D, Kelley AC, Ramakrishnan V: Insights into substrate stabilization from snapshots of the peptidyl transferase center of the intact 70S ribosome. *Nat Struct Mol Biol* 2009; **16:** 528-533.

10 Tourigny DS, Fernandez IS, Kelley AC, Ramakrishnan V: Elongation factor G bound to the ribosome in an intermediate state of translocation. *Science* 2013; **340:** 1235490.

11 Ben-Shem A, Garreau de Loubresse N, Melnikov S, Jenner L, Yusupova G, Yusupov M: The structure of the eukaryotic ribosome at 3.0 A resolution. *Science* 2011; **334:** 1524-1529.
