# Supplementary material for: The presence of highly disruptive 16S rRNA mutations in clinical samples indicates a wider role for mutations of the mitochondrial ribosome in human disease
Source: Mitochondrion. 2015 Nov;25:17–27. doi: 10.1016/j.mito.2015.08.004 (PMC4665369; doi:10.1016/j.mito.2015.08.004)
Supplement: Description of mutations analyzed in this work. [file mmc2.pdf]

Source	Reported patient/ Cell line	mtDNA mutation	rRNA mutation	Reported associated clinical symptoms	Tissue	mtDNA mutation status	Haplotype, ethnicity
[1]		**m.1704T>C**	**34U>C**	Cytochrome c oxidase deficiency	urothelial cell patch	nd	nd
[2]	9T	**m.1792G>A**	**122G>A**	prostate cancer	Tumor	nd	nd
[3]	Patient 5 (crypt 1–)	**m.1831G>A**	**161G>A**		COX-deficient colonic crypt	Heteroplasmic (30%)	nd
[4]	AL4.3 cybrid cell line	**m.1843T>C**	**173U>C**	respiratory deficiency	cell line	61% homoplasmy	
[5]	31673	**m.1886G>A**	**216G>A**	combined complex I + IV deficiency, Mitochondrial Myopathy	muscle		
[6]		**m.1892A>G**	**222A>G**	young obese adult	blood	Homoplasmic	nd
[7]		**m.1905C>A**	**235C>A**		COX-deficient colonic crypt	Homoplasmic	nd
[8]	510	**m.1913G>A**	**243G>A**	breast cancer	Breast cancer samples and corresponding nontumorous breast tissue	Homoplasmic	nd
[9]	Sample 1177	**m.1923C>T**	**253C>U**	Renal carcinoma	Fresh tumor and normal kidney parenchymal tissues	Heteroplasmic (50%)	nd
[4]	AL4.3 cybrid cell line	**m.1940A>G**	**270A>G**	respiratory deficiency	cell line	61% homoplasmy	
[10]	OV30	**m.1952T>C**	**282U>C**		primary ovarian carcinomas and matched normal tissues		
[11]	Tumor V451	**1967T>C**	**297U>C**	human colorectal cancer	normal cells, primary tumours, and tumour cell lines from the same patients	~50%	nd
[12]	Sample 3538	**m.1990G>A**	**320G>A**	squamous cell cancers of the head and neck	tumor tissue and matched normal specimens	Heteroplasmic	nd
[12]	Sample 2818	**m.2004G>A**	**334G>A**	squamous cell cancers of the head and neck	tumor tissue and matched normal specimens	Heteroplasmic	nd
[13]	Tumor 7	**m.2004G>A**	**334G>A**	head and neck squamous cell carcinoma	tumors and matched leukocyte DNA samples	nd	nd
[14]	Sample PCA003	**m.2007T>C**	**337U>C**	Prostate Cancer	benign and cancerous tissue	Heteroplasmic (20%)	H1c
[15]	Panc1	**m.2015G>A**	**345G>A**	pancreatic cancer	pancreatic cancer cell line	Homoplasmic	nd
[12]	Sample 1680	**m.2069T>G**	**399U>G**	squamous cell cancers of the head and neck	tumor tissue and matched normal specimens	Heteroplasmic	nd
[16]	P5	**m.2074delA**	**404delA**	Leigh’s disease	blood	nd	nd
[6]		**m.2079C>T**	**409C>U**	young obese adult	blood	Homoplasmic	nd
[12]	Sample 1680	**m.2083T>G**	**413U>G**	squamous cell cancers of the head and neck	tumor tissue and matched normal specimens	Heteroplasmic	nd

[14]	PCA012	**m.2119 3T-->4T**	**449 3T-->4T**	Prostate Cancer	benign and cancerous tissue	Heteroplasmic (50%)	T2
[17]	C085	**m.2121G>A**	**451G>A**	esophageal cancer	cancerous and adjacent normal tissues	Heteroplasmic	A
[18]	GC3	**m.2148 2149dupAG**	**478-479dupAG**	gastric cancer	gastric cancer tissues and the corresponding adjacent normal tissues	Heteroplasmic	F2a
[14]	PCA005	**m.2150 delT**	**480U del**	Prostate Cancer	benign and cancerous tissue	Heteroplasmic (10%)	H3
[14]	PCA005	**m.2151 delA**	**481A del**	Prostate Cancer	benign and cancerous tissue	Heteroplasmic (10%)	H3
this work		**m.2221C>T**	**551C>U**	Mutation found in an adult patient with unexplained optic atrophy; common LHON mutations excluded	blood	Apparent homoplasmy	
[19]	#078	**m.2238A>G**	**568A>G**	hearing impairment associated with the 12S rRNA T1095C mutation	blood	Homoplasmic	nd
[20]	patient no. 6	**m.2275T>C**	**605U>C**	early stage breast cancer	breast cancerous tissues, corresponding paracancerous normal tissues, and distant normal tissues	Near homoplasmic	B5a
[3]	Patient 2 (crypt 1-)	**m.2275T>C**	**605U>C**		COX-deficient colonic crypt	Heteroplasmic (40%)	nd
[11]	Tumor V410	**m.2299T>A**	**629U>A**	human colorectal cancer	normal cells, primary tumours, and tumour cell lines from the same patients	Heteroplasmic (~50%)	nd
[21]	Patient 6 (cell 8)	**m.2347C>T**	**677C>U**		COX-deficient colonocyte	Heteroplasmic (50%)	nd
[14]	PCA003	**m.2545T>C**	**875U>C**	Prostate Cancer	benign and cancerous tissue	Heteroplasmic (20%)	H1c
[6]		**m.2550A>T**	**880A>U**	young obese adult	blood	Homoplasmic	nd
[7]	Patient 1 (cell 1)	**m.2559T>G**	**889U>G**		COX-deficient colonocyte	Heteroplasmic (50%)	nd
[22]		**m.2593G>A**	**923G>A**		COX-deficient gastric unit		
[12]	Sample 2704	**m.2618T>C**	**948U>C**	squamous cell cancers of the head and neck	tumor tissue and matched normal specimens	Heteroplasmic	nd
[3]	Patient 2 (crypt 7-)	**m.2643G>C**	**973G>C**		COX-deficient colonic crypt	Heteroplasmic (90%)	nd
[23]	MMDB_OCT16S006P	**m.2644A>G**	**974A>G**	Oral Cancer	blood	nd	nd
[24]	898	**m.2664T>C**	**994U>C**	Lung cancer	Tumor and paired blood samples	Homoplasmic	nd
[25]	B14	**m.2680C>T**	**1010C>U**	Breast cancer	paired-tumor and adjacent normal tissue	Heteroplasmic	U4
[26]	2	**m.2681G>A**	**1011G>A**	Follicular thyroid carcinoma	paired-tumor and adjacent normal tissue	Heteroplasmic	nd

[27]	Case 10	**m.2698G>A**	**1028G>A**	squamous cell cancer	Primary pancreatic tumour and matched normal tissue	Heteroplasmic	nd
this work		**m.2784A>C**	**1114A>C**	Child from Irish family with history of SID and developmental delay; some evidence of low I and possibly IV; mutation homoplasmic in blood and muscle, also in blood from unaffected brother – unlikely pathogenic role	Blood and Muscle	Homplasmic	
[28]	Patient 2	**m.2790 InsT**	**1120 InsT**	Congenital Glaucoma	nd	nd	nd
[15]	Xenograft PX17	**m.2805A>T**	**1135A>U**	pancreatic cancer	xenografted primary pancreatic adenocarcinoma	Homoplasmic	nd
[3]	Patient 1 (crypt 4–)	**m.2815G>A**	**1145G>A**		COX-deficient colonic crypt	Heteroplasmic (60%)	nd
[1]		**m.2815G>A**	**1145G>A**	Cytochrome c oxidase deficiency	urothelial cell patch	Homoplasmic	nd
[7]	Patient 2 (cell 9)	**m.2816G>A**	**1146G>A**		COX-deficient colonocyte	Homoplasmic	nd
[29]	90% percent of the studied Dupuytren’s disease patients	**m.2839C>A**	**1169C>A**	Dupuytren’s disease	blood	Heteroplasmic	nd
this work		**m.2884C>T**	**1214C>U**	maternal family history of deafness/SNHL		Heteroplasmic	
[30]	Patient 32	**m.2923G>A**	**1253G>A**	prostate cancer	Areas of benign epithelium and adenocarcinoma	Homoplasmic	nd
this work		**m.2953T>C**	**1283U>C**	Adult patient (21 years old) with ophthalmoparesis, RP, short stature, muscle weakness; mtDNA deletions previously excluded	muscle	Apparently homoplasmic in muscle	
[31]	Patient 8	**m.2998T>C**	**1328U>C**	Breast cancer	cancerous and corresponding normal breast tissue	Heteroplasmic	K1a1b1
[32]	Sample 22	**m.2998 InsT**	**1328 InsU**	Lung cancer	tumor and paired normal tissue	Homoplasmic	nd
[3]	Patient 2 (crypt 6–)	**m.3014G>A**	**1344G>A**		COX-deficient colonic crypt	Homoplasmic	nd
[27]	Case 10	**m.3032G>A**	**1362G>A**	pancreatic cancer	Primary pancreatic tumour and matched normal tissue	Heteroplasmic	nd
[24]	Patient 1127	**m.3054G>A**	**1384G>A**	Bladder cancer	Tumor and paired blood samples	Heteroplasmic	nd
this work		**m.3068G>A**	**1398G>A**	70 yr old male with a tubulovillus adenoma of the rectum		Homoplasmic	T

[12]	Sample 2232	**m.3079G>A**	**1409G>A**	squamous cell cancers of the head and neck	tumor tissue and matched normal specimens	Heteroplasmic	nd
[13]	tumor 3	**m.3079G>A**	**1409G>A**	head and neck squamous cell carcinoma	tumors and matched leukocyte DNA samples	nd	nd
[17]	sample C120	**m.3082G>C**	**1412G>C**	esophageal cancer	cancerous and adjacent normal tissues	Heteroplasmic	M9a3
[33]	20-year-old woman with severe myopathy	**m.3090G>A**	**1420G>A**	myopathy	blood cells, frozen skeletal muscle, urine samples, hair follicles, fibroblasts, and epithelial jugal cells	almost homoplasmic in muscle, 50% heteroplasmic in urine sample. Absent in fibroblast, jugal cells, hair follicles, and in blood cells	nd
[34]	Patient II-2	**m.3093C>G**	**1423C>G**	MELAS syndrome, diabetes mellitus, hyperthyroidism and cardiomyopathy	muscle	Heteroplasmic (51%)	nd
[3]	Patient 2 (crypt 2-)	**m.3094G>A**	**1424G>A**		COX-deficient colonic crypt	heteroplasmic (40%)	nd
[21]	Patient 4 (cell 8)	**m.3103C>T**	**1433C>U**		COX-deficient colonocyte	Heteroplasmic (80%)	nd
[35]		**m.3160A>T**	**1490A>U**	Sporadic Parathyroid Adenoma	Tumor	nd	nd
this work		**m.3205C>T**	**1535C>U**	Child with isolated complex I deficiency; Pakistani origin, consanguineous parents. Homozygous, pathogenic NDUFS1 variant.		Homoplasmic	
