# Supplementary material for: The presence of highly disruptive 16S rRNA mutations in clinical samples indicates a wider role for mutations of the mitochondrial ribosome in human disease
Source: Mitochondrion. 2015 Nov;25:17–27. doi: 10.1016/j.mito.2015.08.004 (PMC4665369; doi:10.1016/j.mito.2015.08.004)
Supplement: Description of structures used in this work. [file mmc3.pdf]

| RCSB Title | Accession codes: SSU, *LSU* | Ribosomal composition | rotation state | mRNA | tRNA | other ligands | Res. (Å) | Refinement Parameters | Organism | Phylogenetic Domain | Ref | PubMed ID |
|---|---|---|---|---|---|---|---|---|---|---|---|---|
| Structure of the large ribosomal subunit from human mitochondria | ***3J7Y*** | LSU | NA | N | N | N | 3.4 | NA | *Homo sapiens* | Mitochondrial | [1] | 25278503 |
| 39S large subunit of the porcine mitochondrial ribosome | ***4CE4*** | LSU | NA | N | N | N | 4.9 | NA | *Sus scrofa* | Mitochondrial | [2] | 24362565 |
| Cryo-EM structure of the small subunit of the mammalian mitochondrial ribosome. | **3J6V** | SSU | NA | N | N | N | 7 | NA | *Bos taurus* | Mitochondrial | [3] | 24799711 |
| Structure of the yeast mitochondrial large ribosomal subunit. | ***1VW3*** | LSU | NA | N | N | N | 3.2 | NA | *Saccharomyces cerevisiae* | Mitochondrial | [4] | 24675956 |
| The re-refined crystal structure of the Haloarcula marismortui large ribosomal subunit at 2.4 Angstrom resolution: more complete structure of the L7/L12 and L1 stalk, L5 and LX proteins | ***4HUB*** | LSU | NA | N | none | none | 2.4 | R-Value: 0.166 (obs.) R-Free: 0.206 | *Haloarcula marismortui* | Archaea | [5] | 23695244 |
| Control of ribosomal subunit rotation by elongation factor G | ***4KIX*** **4KIY** | SSU/LSU | ratcheted | N | none | EFG, viomycin | 2.9 | R-Value: 0.223 (work) R-Free: 0.271 | *Escherichia coli* | Bacteria | [6] | 23812721 |
| Structure of the *Thermus thermophilus* 70S ribosome complexed with mRNA, tRNA and paromomycin | **2J00** ***2J01*** | SSU/LSU | non-ratcheted | Y | A(ASL[%]), P, E | paromomycin | 2.8 | R-Value: 0.271 (obs.) R-Free: 0.313 | *Thermus thermophilus* | Bacteria | [7] | 16959973 |
| The structure of the ribosome with Elongation Factor G trapped in the post-translocational state | **2WRI** ***2WRJ*** **2WRK** ***2WRL*** | SSU/LSU | non-ratcheted | Y | P, E | EFG-fusidic acid | 3.60 | R-Value: 0.227 (obs.) R-Free: 0.260 | *Thermus thermophilus* | Bacteria | [8] | 19833919 |
| Structure of the *Thermus thermophilus* 70S ribosome in complex with mRNA, paromomycin, acylated A- and P-site tRNAs, and E-site tRNA | **2WDK** ***2WDL*** | SSU/LSU | non-ratcheted | Y | aa-A[&],aa-P[&],E | paromomycin | 3.5 | R-Value: 0.208 (obs.) R-Free: 0.256 | *Thermus thermophilus* | Bacteria | [9] | 19363482 |
| Crystal structure of the ribosome bound to elongation factor G in the guanosine triphosphatase state | **4JUW** ***4JUX*** | SSU/LSU | ratcheted | Y | P/E hybrid state tRNA | EF-G, GDPCP | 2.86 | R-Value: 0.220 (obs.) R-Free: 0.250 | Thermus thermophilus | Bacteria | [10] | 19363482 |
| The structure of the eukaryotic ribosome at 3.0 a resolution | **3U5B** **3U5C** ***3U5D*** ***3U5E*** | SSU/LSU | ratcheted | N | none | none | 3.0 | R-Value: 0.182 (obs.) R-Free: 0.228 | *Saccharomyces cerevisiae* | Eucarya | [11] | 22096102 |

% A(ASL) = A-site anticodon stem loop[7].

& aa = amynoacylated
